# Patient-reported outcomes in patients with primary immunodeficiency diseases in Japan: baseline results from a prospective observational study

**DOI:** 10.3389/fimmu.2023.1244250

**Published:** 2023-09-27

**Authors:** Hirokazu Kanegane, Masataka Ishimura, Toshinao Kawai, Satoshi Okada, Nobuaki Okamatsu, Madoka Go, Shinichi Noto

**Affiliations:** ^1^ Department of Child Health and Development, Graduate School of Medical and Dental Sciences, Tokyo Medical and Dental University (TMDU), Tokyo, Japan; ^2^ Department of Pediatrics, Graduate School of Medical Sciences, Kyushu University, Fukuoka, Japan; ^3^ Division of Immunology, National Center for Child Health and Development, Tokyo, Japan; ^4^ Department of Pediatrics, Graduate School of Biomedical and Health Sciences, Hiroshima University, Hiroshima, Japan; ^5^ Medical Franchise Plasma-Derived Therapies, Japan Medical Office, Takeda Pharmaceutical Company Limited, Tokyo, Japan; ^6^ Rare Disease, Evidence Generation & Outcomes Research, Japan Medical Office, Takeda Pharmaceutical Company Limited, Tokyo, Japan; ^7^ Department of Rehabilitation, Niigata University of Health and Welfare, Niigata, Japan

**Keywords:** 36-Item Short Form Health Survey (SF-36), activities of daily living, immunoglobulin replacement therapy, Japan, patient reported outcome measures, primary immunodeficiency diseases, quality of life, Work Productivity and Activity Impairment (WPAI) Questionnaire

## Abstract

**Introduction:**

Primary immunodeficiency diseases (PIDs) are rare inherited diseases resulting in impaired immunity. People with PID experience lower health-related quality of life (HR-QOL) and disease-related burdens in daily activities. This ongoing, prospective observational study aims to evaluate disease activity, treatment status, treatment-related burden, daily activities, and HR-QOL in patients with PID in Japan over a 1-year period. In this interim report (database lock: July 29, 2022), we present baseline results.

**Methods:**

Participants were enrolled between November 2021 and May 2022; data were collected four times/year per participant until May 2023 using an online electronic patient-reported outcomes system. Patients with PID and healthy volunteers aged ≥12 years, residing in Japan, and with access to a smartphone were eligible. HR-QOL (primary endpoint) was assessed by the EuroQol-5 Dimensions-5 Levels (EQ-5D-5L) and the Medical Outcomes Study 36-Item Short Form Health Survey (SF-36). Work productivity was assessed by the Work Productivity and Activity Impairment (WPAI) Questionnaire. Other aspects of PID and burden were assessed with a new questionnaire developed in-house. The study is registered at the University hospital Medical Information Network clinical trials registry (UMIN000045622).

**Results:**

The full interim analysis set comprised 71 patients with PID and 47 healthy volunteers. The most common International Union of Immunological Societies PID category was primary antibody deficiency (56.3% of patients). Complications were common, especially recurrent respiratory tract infections (63.4%). Most patients with PID were treated with immunoglobulin replacement therapy (73.2%); 22.4% of these patients had serum immunoglobulin levels <700 mg/dL. Among patients who did not undergo hematopoietic cell transplantation, EQ-5D-5L (n=67) and SF-36 (n=59) Physical and Mental Component Summary scores were significantly lower than in healthy volunteers (p < 0.001). WPAI absenteeism, work productivity loss, and activity impairment scores were significantly lower in 42 working patients with PID than in 37 working healthy volunteers (p < 0.05). Other results indicated that patients with PID experience substantial burdens related to medical visits, expenses, work, and daily activities.

**Discussion:**

This interim analysis confirms that patients with PID in Japan have lower HR-QOL and work productivity compared with healthy individuals and experience substantial limitations and burdens in their daily lives.

## 1 Introduction

Primary immunodeficiency disease (PID) refers to inherited diseases caused by genetic variants of immunoregulatory proteins, resulting in impairment of the immune system (1, 2). PID is primarily characterized by increased susceptibility to infections but can also result in autoimmune diseases, autoinflammatory diseases, and malignancies (1, 2). PID ranges from mild forms that do not require ongoing treatment to critically severe forms, such as severe combined immunodeficiency, that require immediate life-saving intervention. Immunoglobulin replacement therapy (IgRT) is the standard supportive treatment for most patients with antibody deficiencies, although more definitive options, such as allogeneic hematopoietic cell transplantation (HCT), may be suitable for some patients (3, 4). Prophylactic antibiotic and antifungal medications are also often prescribed (4).

PID is considered a rare disease, occurring in 1 per 10,000 to 1 per 50,000 live births, although recent evidence suggests the prevalence may be much higher (5). In Japan, PID occurs in 2.2 per 100,000 persons and is designated as an intractable disease eligible for public assistance (6). To date, nearly 500 PID disorders have been described; these disorders have been classified by the International Union of Immunological Societies (IUIS) based on phenotypic and genetic characteristics (2). In Japan, the most common IUIS categories are primary antibody deficiency (40%), congenital phagocyte dysfunction or defect (19%), and characteristic syndromes associated with immunodeficiency (16%); the most common clinical forms of PID are X-linked agammaglobulinemia (XLA; 15%), chronic granulomatosis disease (12%), and common variable immunodeficiency (CVID; 11%) (7).

People with PID often experience lower health-related quality of life (HR-QOL) and limitations on their daily activities compared with healthy individuals (8–11). Ongoing symptoms, complications, and recurrent infections, as well as physical and financial burdens related to treatment, reduce HR-QOL. In addition, daily activities, particularly those related to school and work, are often substantially limited by PID. A survey conducted by the European Federation of Pharmaceutical Industries and Associations Japan (EFPIA Japan) indicated that patients with PID in Japan experience PID-related burdens, limitations on daily activities, and frequent complications and infections despite treatment (12). Although the survey provided insights into the effects of PID on patients in Japan, it did not evaluate HR-QOL using validated instruments. Moreover, the survey did not follow patients longitudinally to determine the effects of seasonal changes in the environment, which are known to affect the incidence of infections, including in Japan (13–15).

The aim of this prospective observational study is to evaluate disease activity, treatment status, treatment-related burden, daily activities, and HR-QOL in patients with PID in Japan over a 1-year period. In this interim report, we present the baseline results, which provide an extensive overview of the status of patients with PID in Japan and the effects of PID on their HR-QOL.

## 2 Materials and methods

### 2.1 Study design

This was a prospective, non-interventional observational study conducted in Japan. Participants were not required to visit study sites; instead, all data were collected using an online electronic patient-reported outcomes (ePRO) system (3H P-Guardian, developed by 3H Clinical Trial Inc., Tokyo, Japan). The ePRO system was designed in accordance with the Japanese Ministry of Health, Labour and Welfare “Guideline on the Use of Electronic Records and Electronic Signatures in Submission for Approvals and Licenses of Medical Products”, and with Information Security Management System (IEC/ISO 27001) and Privacy Information Management System (IEC/ISO 27701) standards. To protect the privacy of participants, their names were not collected, but an email address was obtained to send vouchers as payment for participating in the study. Participants were enrolled between November 2021 and May 2022, and data were collected for 1 year after enrollment until May 2023. Participants aged ≥16 years at enrollment were followed for 365 days after enrollment and answered the same questions in the ePRO four times (every 3 months, ie, once per season) during the study. Because the HR-QOL instruments used in this study are validated for use in adults aged ≥16 years, participants aged 12 to <16 years were considered to have completed the study after answering the questions just one time, such that only baseline data are available for these adolescents. This interim report includes data collected up to July 29, 2022. The study protocol, informed consent form, and study questionnaire were approved by the Ethics Committee of the Japan Conference of Clinical Research (a non-profit organization). The study is registered at the University hospital Medical Information Network clinical trials registry (ID: UMIN000045622). Written informed consent was provided online by all participants or their legal representatives before enrollment.

### 2.2 Study population

Patients with PID and healthy volunteers who were aged ≥12 years, resided in Japan, and possessed (or whose legal representative possessed) a smartphone were eligible for enrollment. In addition to a PID diagnosis, patients with PID were registered at or were referred from PID Tsubasa-No-Kai (npo-pidtsubasa.org), a non-profit patient association, or were referred by a physician affiliated with the Japanese Society for Immunodeficiency and Autoinflammatory Diseases. Patients with PID who had been diagnosed with an inherited autoinflammatory disease, such as familial Mediterranean fever, were excluded. Healthy volunteers were recruited from a volunteer database curated by 3H Clinical Trial, Inc. and were excluded if they had any of the following: type 2 diabetes, hypertension, dyslipidemia, liver disease, renal disease, thyroid disease, cardiac disease, adrenal disease, any other metabolic disorder, any mental disease such as depression, any physical symptoms that interfered with daily activities, regular medication use or clinic visits for any disease (except seasonal allergic diseases), history of any severe medical condition, drug or food allergy, or a family history of PID. All potential participants could access a research-designed website that explained the study; those who were interested in participating provided informed consent before proceeding to answer screening questions to assess eligibility. Participants who met eligibility criteria were enrolled and proceeded to answer questions within the ePRO system; those who did not meet eligibility criteria exited the system and no data were collected.

### 2.3 Outcome measurements

The primary endpoint is the HR-QOL of patients with PID as assessed by two validated instruments, the EuroQol-5 Dimensions-5 Levels (EQ-5D-5L) and the Medical Outcomes Study 36-Item Short Form Health Survey (SF-36). The EQ-5D-5L assesses five dimensions of HR-QOL (mobility, self-care, usual activities, pain/discomfort, and anxiety/depression), each with five possible responses; in addition, participants rate their overall health from 0 to 100 on a visual analog scale (VAS) (16). The SF-36 assesses physical functioning, bodily pain, role limitations due to physical health problems, role limitations due to personal or emotional problems, emotional well-being, social functioning, energy/fatigue, and general health perceptions (17). Responses are collated into Physical Component Summary (PCS) and Mental Component Summary (MCS) scores. For this study, participants answered SF-36 items in reference to the previous 4 weeks. Validated Japanese versions of the EQ-5D-5L (18) and SF-36 (19) were used.

Secondary endpoints include: 1) HR-QOL (assessed by EQ-5D-5L and SF-36) of patients with PID compared with healthy volunteers and/or national reference values (SF-36 only; (http://www.qualitest.jp/faq/faq_sftool.html); 2) work productivity in participants (patients with PID and healthy volunteers) who are employed, assessed with a certified Japanese translation (available at http://www.reillyassociates.net/WPAI_Translations.html) of the Work Productivity and Activity Impairment (WPAI) Questionnaire (General Health version 2.0) (20), which assesses absenteeism, presenteeism, total work productivity impairment (absenteeism plus presenteeism), and total activity impairment outside the workplace; and 3) burdens in daily life related to infections/symptoms (within the past 3 months), treatments (expected in the next 3 months), or prevention of infections/symptoms (expected in the next 3 months) in patients with PID, using a questionnaire developed specifically for this study (**Supplementary Table 1**).

Demographic information (age, sex) was collected in all participants. Information on disease background, visits to medical institutions, treatments for PID, work-related information, and social support and expenses was collected only in patients with PID (**Supplementary Table 1**).

### 2.4 Statistical analysis

As PID is a rare disease, recruitment of a large number of patients may be difficult; however, based on the sample size of the EFPIA Japan survey (N=165) (12), we aimed to enroll 150 patients with PID in this study. We expected that 50 healthy volunteers could be recruited from the 3H Clinical Trial volunteer database during the study period. Sample size was not based on power calculations.

The full analysis set (FAS) consists of all enrolled participants who completed the HR-QOL questionnaires at least once; the per-protocol set (PPS) excludes patients with PID who only answered one or two of the PID disease-related questions and healthy volunteers who responded to HR-QOL questions but could not be considered healthy at the time they answered the questionnaire because of acute illness. For this interim analysis, the PPS is the primary set, and the FAS is used for reference. Demographic data were analyzed for the FAS and the PPS in patients with PID and in healthy volunteers. Disease-related data (background, medical visits, treatments, limitations, complications, work, etc.) were evaluated in patients with PID. HR-QOL and WPAI were assessed in patients with PID and healthy volunteers. For this interim analysis, HR-QOL and WPAI were evaluated in the subgroup of patients with PID who had not undergone HCT (non-HCT PID subgroup). Patients who underwent HCT were excluded from this subgroup analysis as they were considered to have very different clinical backgrounds from patients who had not undergone HCT. Categorical variables are presented as n (%), and continuous variables are presented as summary statistics (mean and standard deviation [SD]; median and range). WPAI data are presented as scores for individual participants who answered all four WPAI questions.

EQ-5D-5L and SF-36 scores were compared statistically between the non-HCT PID subgroup and healthy volunteers using an unpaired t-test. SF-36 scores were also compared between the non-HCT PID subgroup and national reference values based on a sample of 3286 Japanese people, which included people with chronic conditions (http://www.qualitest.jp/faq/faq_sftool.html), using a one-group t-test. WPAI scores were compared between the non-HCT PID subgroup and healthy volunteers using the non-parametric Mann-Whitney U test. Statistical tests were conducted at a two-sided significance level of 0.05. SAS 9.4 or higher was used for all analyses (SAS Institute Inc., Cary, NC, USA).

## 3 Results

### 3.1 Participant flow and demographics

**Figure 1:**
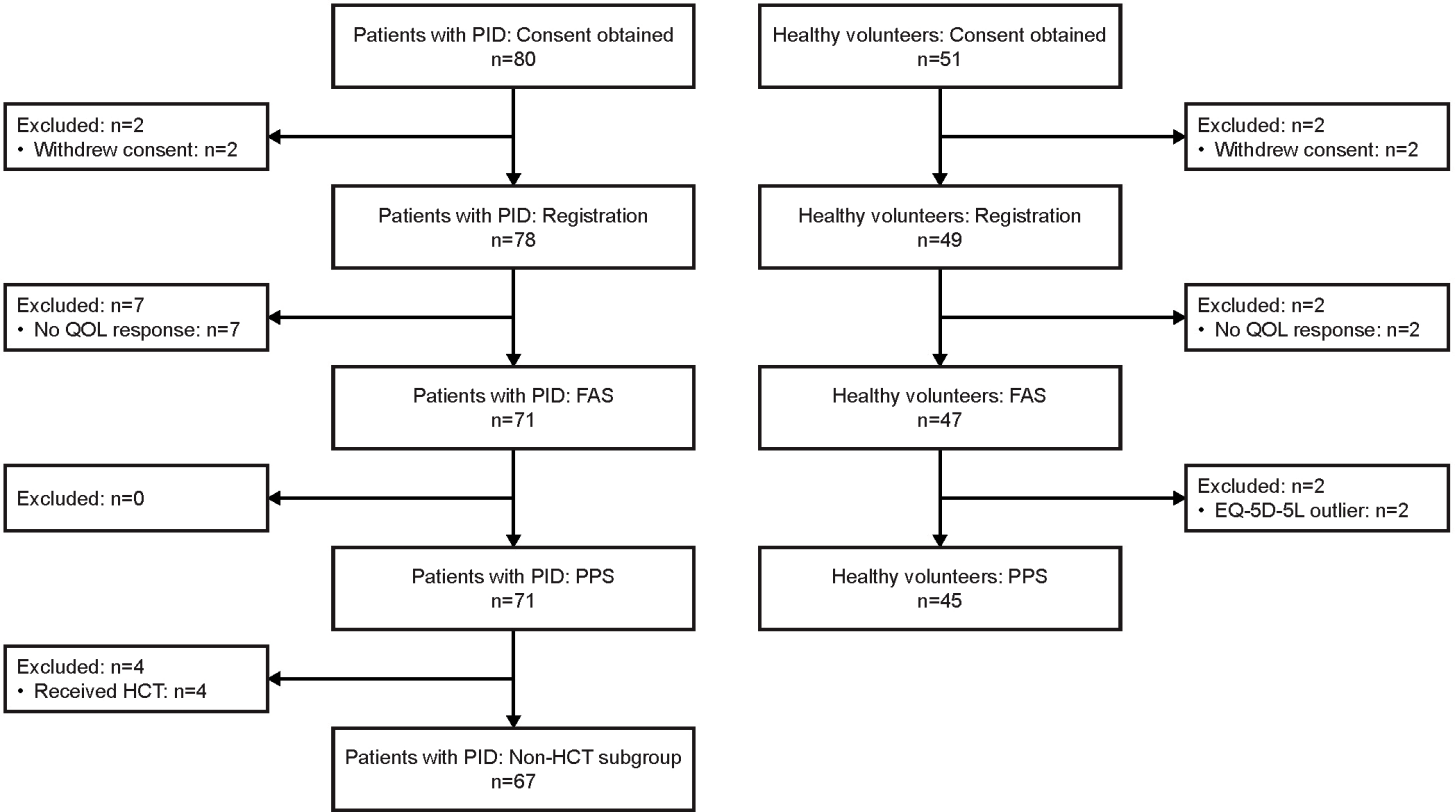
Participant flow diagram. EQ-5D-5L, EuroQol-5 Dimensions-5 Levels; FAS, full analysis set; HCT, hematopoietic cell transplantation; PID, primary immunodeficiency disease; PPS, per-protocol set; QOL, quality of life.

As of the cut-off date for this interim analysis (July 29, 2022), consent was obtained from 80 patients with PID and 51 healthy volunteers (**Figure 1**). Of these, 71 patients with PID were included in the FAS and in the PPS, 47 healthy volunteers were included in the FAS, and 45 healthy volunteers were included in the PPS. Four patients with PID underwent HCT and were excluded from the non-HCT PID subgroup (a subgroup of the PPS). In all analysis groups, approximately 60–65% of participants were male, and the median age was 32–35 years (**Table 1**). Nine (12.7%) patients with PID were ≤15 years old, whereas there were no healthy volunteers this age.

**Table 1:** Demographics of participants.

Variable	Patients with PID (FAS/PPS) (N=71)	Patients with PID (non-HCT subgroup) (N=67)	Healthy volunteers (FAS) (N=47)	Healthy volunteers (PPS) (N=45)
Sex, n (%)				
Male	43 (60.6)	40 (59.7)	31 (66.0)	29 (64.4)
Female	28 (39.4)	27 (40.3)	16 (34.0)	16 (35.6)
Age, years				
Mean (SD)	34.7 (13.8)	35.0 (13.9)	35.6 (12.2)	35.0 (11.8)
Median (min., max.)	35.0 (12, 63)	35.0 (12, 63)	33.0 (17, 62)	32.0 (17, 62)
Age category, n (%)				
≤12 years	2 (2.8)	2 (3.0)	0 (0)	0 (0)
13–15 years	7 (9.9)	7 (10.4)	0 (0)	0 (0)
16–20 years	3 (4.2)	3 (4.5)	7 (14.9)	7 (15.6)
21–29 years	14 (19.7)	12 (17.9)	8 (17.0)	8 (17.8)
30–39 years	17 (23.9)	15 (22.4)	16 (34.0)	15 (33.3)
40–49 years	17 (23.9)	17 (25.4)	9 (19.1)	9 (20.0)
50–59 years	9 (12.7)	9 (13.4)	4 (8.5)	4 (8.9)
≥60 years	2 (2.8)	2 (3.0)	3 (6.4)	2 (4.4)

FAS, full analysis set; HCT, hematopoietic cell transplantation; max., maximum; min., minimum; PID, primary immunodeficiency disease; PPS, per-protocol set; SD, standard deviation.

### 3.2 Disease characteristics in patients with PID

**Figure 2:**
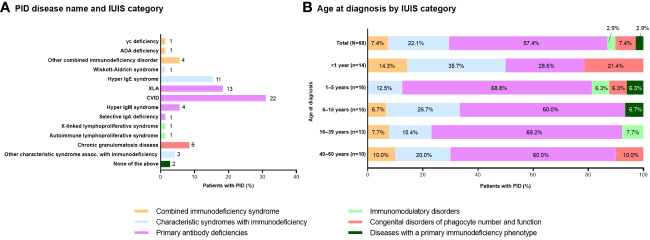
Distribution of patients with PID based on **(A)** disease name and IUIS category (n=71; number of patients with each disease is shown to the right of each column) and **(B)** age at diagnosis by IUIS category (n=68; unknowns excluded). ADA, adenosine deaminase; CVID, common variable immunodeficiency; Ig, immunoglobulin; IUIS, International Union of Immunological Societies; PID, primary immunodeficiency disease; XLA, X-linked agammaglobulinemia.

The most common IUIS category of PID was primary antibody deficiencies (56.3% of patients), particularly CVID (31.0%) and XLA (18.3%) (**Figures 2A**, **2B**). PID was diagnosed at a range of ages from infancy to adulthood (**Figure 2B**). Age at diagnosis was <1 year in 20.6%, 1–5 years in 23.5%, 6–15 years in 22.1%, 16–39 years in 19.1%, and 40–60 years in 14.7% of patients. PID diagnosis was based on symptoms (n=61; 85.9%) or a family history (n=10; 14.1%). Median (minimum, maximum) time to diagnosis was 10 (1, 348) months.

**Figure 3:**
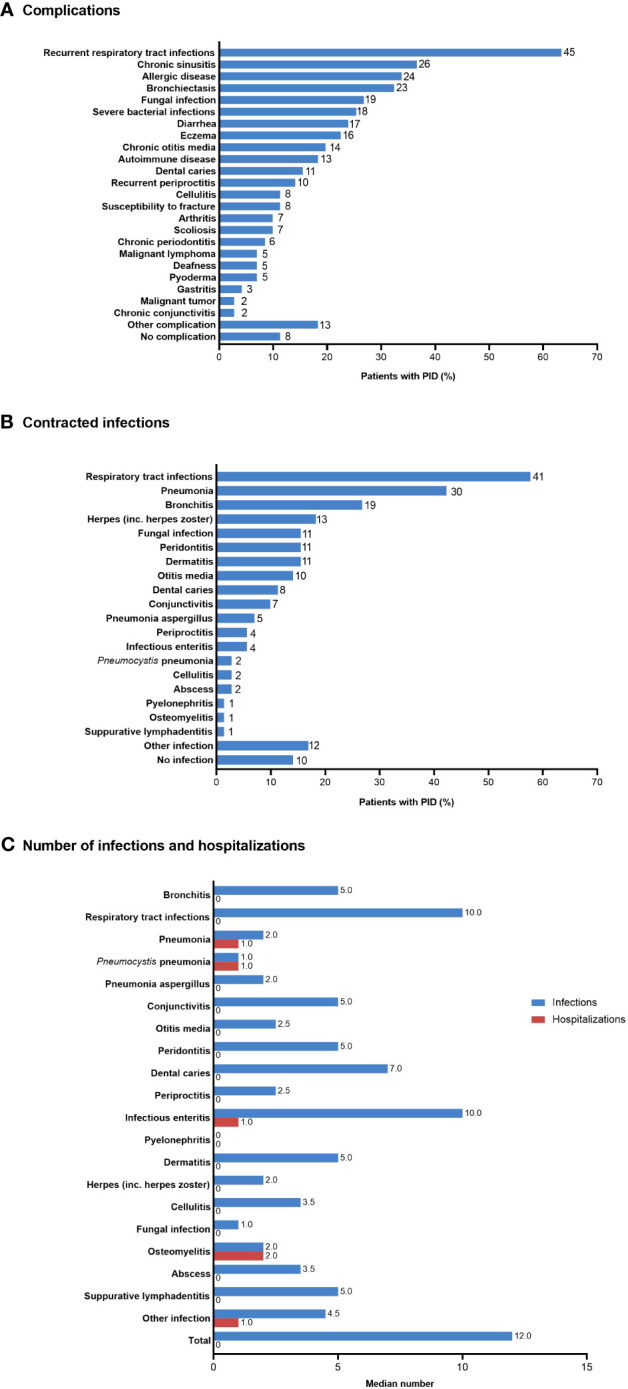
**(A)** Complications, **(B)** infections contracted during the previous 5 years, and **(C)** median number of infections and hospitalizations during the previous 5 years in patients with PID (n=71). In **(A)** and **(B)**, the number of patients is shown to the right of each column; multiple responses from individual patients were possible. PID, primary immunodeficiency disease.

Most patients with PID experienced one or more complications, most commonly recurrent respiratory tract infections (n=45; 63.4%) (**Figure 3A**). Similarly, the most common infections contracted during the previous 5 years were respiratory tract infections (n=41; 57.7%), pneumonia (n=30; 42.3%), and bronchitis (n=19; 26.8%) (**Figure 3B**). The median number of infections during the previous 5 years was 12, with respiratory tract infections and infectious enteritis being the most frequent (median of 10 for each infection type) (**Figure 3C**). Hospitalizations during the previous 5 years were observed for osteomyelitis (median 2), pneumonia, *Pneumocystis* pneumonia, infectious enteritis, and other infections (median 1 each).

**Figure 4:**
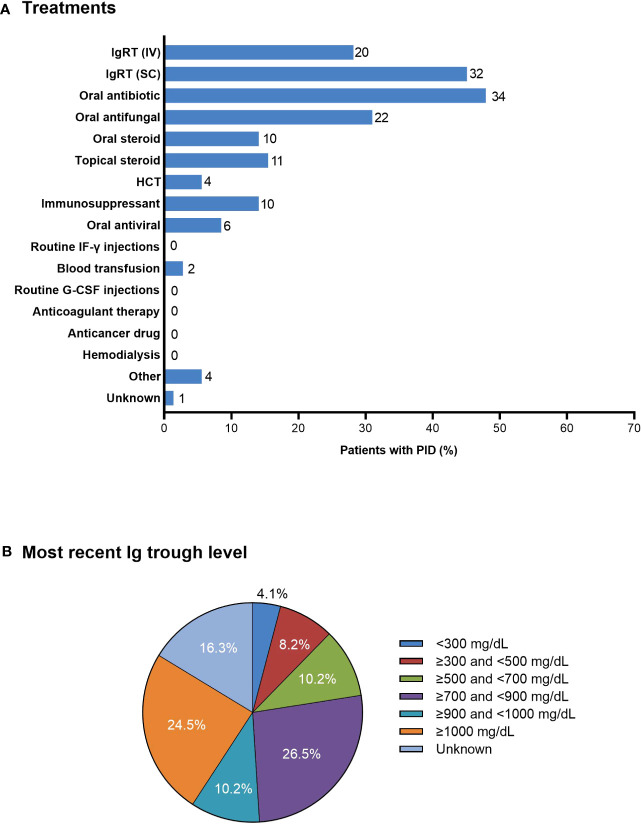
**(A)** Treatments in patients with PID (n=71). The number of patients is shown to the right of each column; multiple responses from individual patients were possible. **(B)** Most recent serum Ig trough levels as reported by patients receiving IV or SC IgRT (n=49). G-CSF, granulocyte colony-stimulating factor; HCT, hematopoietic cell transplantation; IF-γ, interferon-γ; Ig, immunoglobulin; IgRT, immunoglobulin replacement therapy; IV, intravenous; PID, primary immunodeficiency disease; SC, subcutaneous.

Most patients with PID were treated with subcutaneous and/or intravenous IgRT (n=32; 45.1% and n=20; 28.2%, respectively), oral antibiotics (n=34; 47.9%), oral antifungal medication (n=22; 31.0%), and/or oral or topical steroids (n=10; 14.1% and n=11; 15.5%, respectively) (**Figure 4A**). Four (5.6%) patients underwent HCT. Of the 49 patients who received IgRT, 29 (59.2%) were aware of their serum IgG levels at the time of their first dose and 32 (65.3%) reported having regular IgG measurements. The most recent serum IgG trough levels were <700 mg/dL and <500 mg/dL in 11 (22.4%) and six (12.2%) patients, respectively; eight (16.3%) patients did not know their most recent serum IgG trough level (**Figure 4B**).

### 3.3 Disease-related activities of daily life

#### 3.3.1 Medical visits

More than half (52.1%) of patients attended more than one medical institution or more than one department within a single medical institution for their treatment and disease management. (**Supplementary Figure 1**) The most common frequency of medical visits was once a month (40.8%). Almost half (47.9%) of patients reported that the time burden of medical visits was very difficult or somewhat difficult. Almost a third (29.6%) of patients spent longer than 5 hours at the medical institution, and 45.1% spent longer than 1 hour traveling to the medical institution; some patients (9.9%) spent ≥5000 Japanese yen (currently ~38 US dollars) on transport per visit. Patients most commonly visited a pediatrics department (39%) and were seen by a specialist physician (73%). For acute symptoms, most patients visited the university or general hospital that they usually visited (42.3% and 29.6%, respectively), although many patients visited a local clinic different from their usual medical institution (28.2%).

#### 3.3.2 Expenses and social support 

Most patients (81.7%) considered expenses related to PID to be a great burden or somewhat of a burden (**Supplementary Figure 2**). However, a similar percentage of patients (76.1%) indicated that all their expenses are subsidized. Approximately 65–70% of patients indicated that a physical disability certificate was necessary or somewhat necessary to cover medical expenses, provide discounted fares, expand opportunities for work, and provide appropriate workplace considerations. However, only nine (13%) patients had a physical disability certificate, which they obtained because of upper limb, lower limb, respiratory, and/or other issues.

#### 3.3.3 Work and school status 

More than half of patients were either fully employed (37%) or students (23%); however, a number were in part-time employment (14%) or were unemployed (11%) (**Supplementary Figure 3**). Among the patients who were <18 years old, two (3% of the PID FAS) were in elementary school, six (8%) were in junior high school, and three (4%) were in high school.

#### 3.3.4 Work-related data

Among the 55 patients with PID who were not students, 25% had problems getting a job at any time, 20% had to resign or take a leave of absence, and 15% had problems passing a job interview because of their PID; only 15% never had any problems with work. (**Supplementary Figure 4**) Although 53% of non-student patients indicated that their workplace made certain accommodations for their disease and were satisfied with their current work situation, 27% indicated that they would prefer a workplace that could better accommodate their disease; 9% of patients felt their symptoms made it too difficult for them to work. Among 13 patients who were homemakers, industrial homeworkers (eg, doing piecework), on leave, or unemployed, most were not working in a job that requires commuting because of their disease. The median age of seven patients who had retired or were on leave was 39 years, and they had been retired or on leave from work for a median of 24 months.

### 3.4 HR-QOL

**Figure 5:**
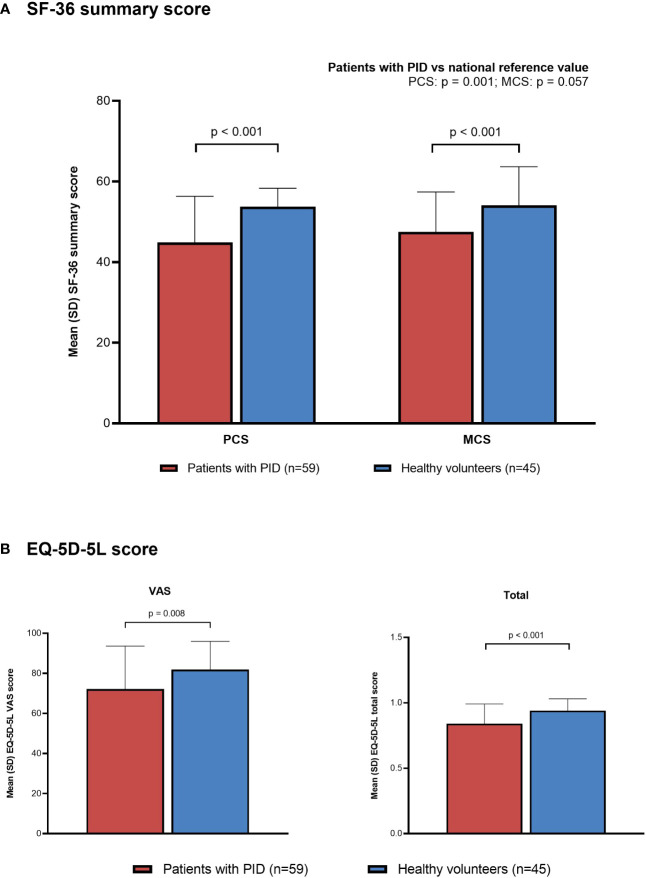
Quality of life in patients with PID (non-HCT subgroup) compared with healthy individuals (PPS). **(A)** SF-36, **(B)** EQ-5D-5L. EQ-5D-5L, EuroQol-5 Dimensions-5 Levels; HCT, hematopoietic cell transplantation; MCS, Mental Component Summary; PCS, Physical Component Summary; PID, primary immunodeficiency disease; PPS, per-protocol set; SD, standard deviation; SF-36, 36-Item Short Form Health Survey; VAS, visual analog scale.

Both SF-36 PCS and MCS summary scores were significantly lower in the non-HCT PID subgroup than in the healthy volunteer group (**Figure 5A**). Mean (SD) summary scores in the non-HCT PID subgroup and healthy volunteer group, respectively, were 44.9 (11.4) vs 53.8 (4.5) for PCS and 47.5 (9.9) vs 54.1 (9.6) for MCS (p < 0.001 for both). Both the PCS and MCS summary scores in the non-HCT PID subgroup were lower than the national reference value; statistical significance was reached for the PCS summary score (p = 0.001) but not for the MCS summary score (p = 0.057). All SF-36 subscales except Mental Health were significantly lower in the non-HCT PID subgroup than in healthy volunteers (mean [SD]: Physical Functioning: 83.5 [20.6] vs 96.6 [6.7], p < 0.001; Role-Physical: 74.3 [24.1] vs 95.0 [8.7], p < 0.001; Bodily Pain: 66.5 [27.2] vs 82.1 [17.3], p = 0.001; General Health: 36.1 [18.6] vs 74.6 [17.0], p < 0.001; Vitality: 51.1 [22.4] vs 62.3 [20.9], p = 0.011; Social Functioning: 74.6 [24.0] vs 91.4 [14.3], p < 0.001; Role-Emotional: 77.4 [20.1] vs 87.2 [17.4], p = 0.010; Mental Health: 69.3 [18.8] vs 74.4 [17.0], p = 0.155) (**Supplementary Figure 5**). SF-36 scores and subscores in the PID PPS group were similar to those in the non-HCT PID subgroup (data not shown).

Both EQ-5D-5L VAS and total scores were also significantly lower in the non-HCT PID subgroup than in the healthy volunteer group (**Figure 5B**). Mean (SD) EQ-5D-5L VAS and total scores in the non-HCT PID subgroup and healthy volunteer group, respectively, were 72.2 (21.4) vs 81.9 (14.0) (p = 0.008) and 0.84 (0.15) vs 0.94 (0.09) (p < 0.001). There were also significant differences in the distribution of responses for the EQ-5D-5L dimensions of mobility (p = 0.008), usual activities (p = 0.022), and pain/discomfort (p = 0.015), with patients in the non-HCT PID subgroup selecting scores that indicate lower HR-QOL compared with healthy volunteers; no significant differences were seen for the dimensions of self-care and anxiety/depression.

### 3.5 Work productivity

**Figure 6:**
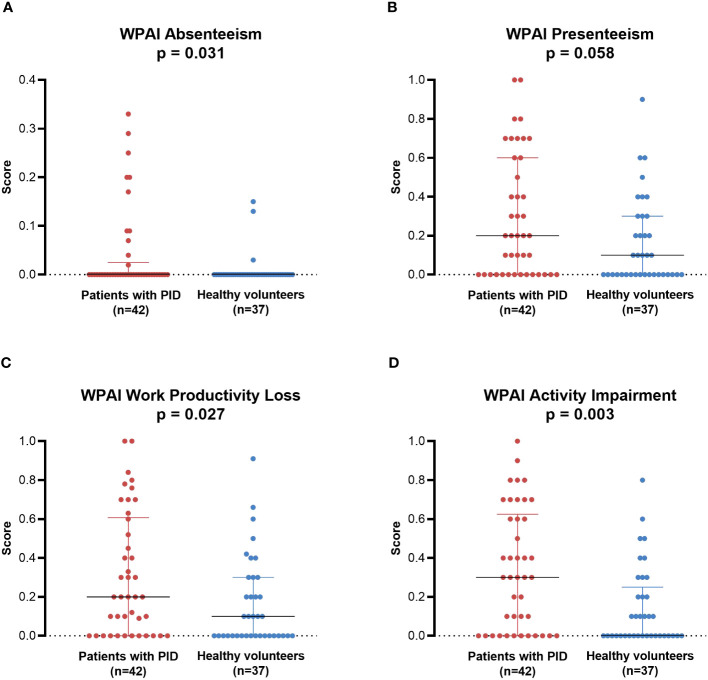
WPAI in individual patients with PID (non-HCT subgroup; n=42) compared with healthy individuals (PPS; n=37). **(A)** Absenteeism, **(B)** Presenteeism, **(C)** Work Productivity Loss, **(D)** Activity Impairment. Lines represent medians; error bars represent interquartile ranges. p-Values are from the Mann-Whitney U test comparing between groups. HCT, hematopoietic cell transplantation; PID, primary immunodeficiency disease; PPS, per-protocol set; WPAI, Work Productivity and Activity Impairment.

Among participants who were currently working and answered all four WPAI questions (non-HCT subgroup, n=42; healthy volunteers, n=37), patients with PID had significantly greater absenteeism (median [minimum, maximum]: 0.000 [0.00, 0.33] vs 0.000 [0.00, 0.15]; p = 0.031), work productivity loss (0.200 [0.00, 1.00] vs 0.100 [0.00, 0.91]; p = 0.027), and activity impairment (0.300 [0.00, 1.00] vs 0.000 [0.00, 0.80]; p = 0.003) compared with healthy volunteers (**Figure 6**). Presenteeism tended to be greater in patients with PID than in healthy volunteers, but the difference was not statistically significant (0.200 [0.00, 1.00] vs 0.100 [0.00, 0.90]; p = 0.058). Similar results were also observed when comparing the PID PPS group (including patients who had undergone HCT; n=44) with healthy volunteers (n=37) (**Supplementary Figure 6**).

### 3.6 Disease-related burdens in daily life

**Figure 7:**
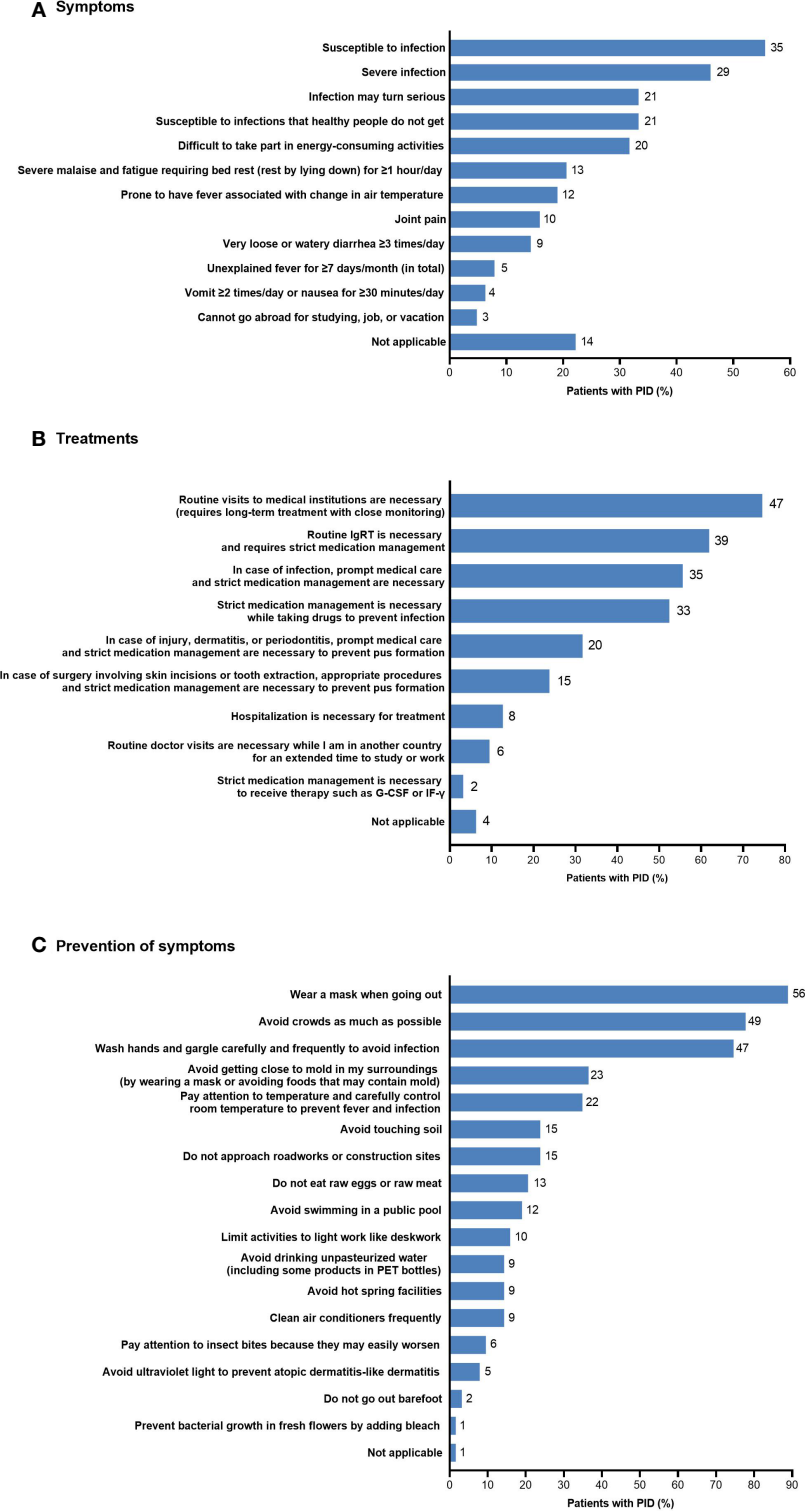
Disease-related burdens in daily life in patients with PID (non-HCT subgroup; n=63) related to **(A)** symptoms, **(B)** treatments, and **(C)** prevention of symptoms. The number of patients is shown to the right of each column; multiple responses from individual patients were possible. G-CSF, granulocyte colony-stimulating factor; HCT, hematopoietic cell transplantation; IF-γ, interferon-γ; IgRT, immunoglobulin replacement therapy; PET, polyethylene terephthalate; PID, primary immunodeficiency disease.

A number of disease-related burdens in daily life related to PID were reported by more than 50% of patients in the non-HCT PID subgroup (n=63) (**Figure 7**). The most common burdens in daily life resulting from disease symptoms during the previous 3 months were susceptibility to infection (n=35; 55.6%) and having a severe infection (n=29; 46.0%). The most common burdens related to future treatments included the need for routine visits to medical institutions for long-term treatment with close monitoring (n=47; 74.6%), the need for routine IgRT with strict medical management (n=39; 61.9%), the need for prompt medical care and strict medication management in case of infection (n=35; 55.6%), and the need for strict medication management while taking drugs to prevent infection (n=33; 52.4%). The most common burdens related to prevention of symptoms included wearing a mask when going out (n=56; 88.9%), avoiding crowds as much as possible (n=49; 77.8%), and washing hands and gargling carefully and frequently (n=47; 74.6%).

## 4 Discussion

This interim analysis of a prospective observational study provides the first comprehensive, quantitative assessment of disease and treatment status, HR-QOL, and disease-related burdens in daily life in patients with PID in Japan. Importantly, this is the first analysis to use validated instruments to evaluate HR-QOL (SF-36, EQ-5D-5L) and work productivity (WPAI) in Japanese patients with PID. The results confirm that patients with PID in Japan have lower HR-QOL and work productivity compared with healthy volunteers and national reference values. In addition, patients with PID experience substantial limitations and disease-related burdens in their daily lives.

Patients with PID in this study were generally similar to those in previous reports from Japan in terms of age, sex, and type of PID (7, 12), suggesting that the study sample was representative of the overall population of Japanese patients with PID. Notably, the median time to PID diagnosis was 10 months, with the longest time being 348 months (29 years), and a large proportion of patients were not diagnosed until late adolescence or adulthood. Although the median time to diagnosis was much shorter than the 4–12 years reported in other countries (21–23), the delay can vary depending on the type of PID and the country’s health system. Delayed diagnosis has previously been identified as problematic in Japan (7, 24). Although efforts have been made in Japan and in other countries to increase awareness among physicians of the “10 warning signs of PID” (24, 25), our results suggest greater knowledge regarding PID is still required.

Most patients received intravenous or subcutaneous IgRT; however, only 65% of patients receiving IgRT indicated that their serum IgG trough levels were measured before every treatment. Furthermore, trough levels were <700 mg/dL in about 22% of patients and <500 mg/dL in about 12% of patients; about 16% of patients did not know their most recent IgG trough level. Although target IgG trough levels vary depending on the type of PID and individual patient needs, most guidelines, including Japanese guidelines, recommend trough levels >700 mg/dL (26–28). IgG trough levels have been shown to inversely correlate with the incidence of pneumonia, with a five-fold difference in pneumonia incidence between trough levels of 500 mg/dL compared with 1000 mg/dL; this provides strong evidence that maintaining sufficient IgG levels helps prevent serious infections (29). However, a recent survey of physicians in Japan with IgRT experience reported that a third (32.8%) considered target IgG trough levels to be 400–500 mg/dL, while a quarter (23.0%) considered 500–700 mg/dL as the target range (24). Together with the results of our study, these findings indicate that the IgG levels of some patients with PID in Japan are lower than needed to provide optimal protection against infection; this is reflected in the high proportion of patients having recurrent infections, some of which required hospitalization. Increased education is needed to help physicians appreciate the relationship between IgG trough levels and infection risk and to understand the importance of providing sufficient IgRT.

HR-QOL, assessed by SF-36 and EQ-5D-5L, was significantly poorer in patients with PID (excluding those who underwent HCT) than in healthy volunteers. Both SF-36 PCS and MCS scores, as well as most subscales, were significantly lower in patients with PID, indicating that PID affects a broad range of aspects of quality of life. Interestingly, no significant difference between groups was observed for the SF-36 Mental Health subscale. Although the reason for this is unclear, it may be related to the COVID-19 pandemic, which was ongoing at the time of the survey and may have affected the mental health of all participants similarly. Both SF-36 MCS and PCS scores in patients with PID were also lower than national reference values, although only the PCS score was statistically significantly different. Likewise, EQ-5D-5L total and VAS scores were significantly lower in patients with PID compared with healthy volunteers. Patients were most greatly affected in the dimensions of mobility, usual activities, and pain/discomfort; aspects of self-care and anxiety/depression were less affected. These results are highly consistent with those of a global study that used the EQ-5D-5L VAS and a shorter version of the SF-36 to measure HR-QOL in patients with PID (9).

To the best of our knowledge, no previous study worldwide has evaluated work productivity in patients with PID, although the WPAI has been used in studies of patients with one specific type of PID, hereditary angioedema, with generally similar levels of impairment as found in our study (30, 31). Our analysis indicates that patients with PID experience greater levels of absenteeism, work productivity loss, and activity impairment than healthy volunteers. In addition, most non-student patients reported that their disease affected their ability to obtain or keep a job. Although about half of workers indicated that their employer made accommodations for their disease, approximately a quarter would prefer more assistance at work. Consistent with this, approximately 65–70% of patients with PID thought that physical disability certificates would help in securing workplace accommodations and greater employment opportunities, as well as reducing financial burdens. Despite this, only 13% of patients had a disability certificate; this may reflect strict conditions for certification that are difficult for patients with PID to meet, as disability certificates are only available if these patients have severe complications (eg, those affecting the upper or lower limbs, respiratory system, etc.). Overall, these results indicate that work productivity is reduced by PID and that better working conditions, including expanded access to disability certification and proactively providing information to employers, may improve the experience of employed patients.

The lower HR-QOL and work productivity seen in patients with PID reflect the extent of the complications, limitations, and burdens of daily life described in this study. The majority of patients in this study reported having multiple, recurrent infections—particularly respiratory infections—as well as other complications. Patients needed to make frequent, time-consuming, and costly medical visits to receive treatment and other medical care. Patients also needed to be diligent in taking precautions against infection, including managing prophylactic treatment, wearing a mask, and avoiding crowds; these limitations were likely to have been especially burdensome during the COVID-19 pandemic. However, despite these limitations, most patients with PID can have an essentially normal social life if they are appropriately treated, such as through maintaining adequate IgG levels. In addition to treatment, patients need enhanced social support to help reduce the physical, psychological, and financial burden of living with PID.

This study assessed a broad range of factors related to PID and its treatment, providing a comprehensive overview of the status of these patients. The study follows patients with PID, as well as healthy volunteers for comparison, for 1 year to determine how HR-QOL is affected by seasonal changes. However, this study does have some limitations. First, because PID is relatively rare, all patients with PID were combined for the analysis of HR-QOL and other outcomes. However, PID disorders are diverse, with different disease status, treatment options, and prognosis, which could affect HR-QOL. Second, our study was not able to analyze HR-QOL related to specific PID disorders or specific treatments. Third, given that the median age of patients with PID was 35 years, there may be recall bias for some background information such as age at diagnosis. Fourth, all data are patient reported, which may affect the accuracy of some data. Fifth, the sample size is relatively small, and although the sample appears to be representative, the results may not exactly reflect the situation of all patients with PID in Japan. Sixth, people with significant, but common, comorbidities were excluded from the healthy control group, and therefore this group represents a population with optimal health. As such, differences in HR-QOL and work productivity between patients with PID and the general population are likely to be smaller than the differences between patients and the healthy control group observed in this study. Moreover, some patients with PID may also have comorbidities that were excluded from the control group but that could contribute to poorer HR-QOL. Finally, as the study was initiated while the COVID-19 pandemic was ongoing (November 2021 to May 2022), the results in both patients with PID and healthy volunteers may be unusual.

In conclusion, this interim analysis of an ongoing observational study has confirmed that patients with PID in Japan have lower HR-QOL and work productivity compared with healthy individuals and experience substantial limitations and burdens in their daily lives. Improved disease management, such as ensuring adequate IgG levels are maintained, increased awareness among physicians, and increased access to social support are needed to improve overall quality of life in patients with PID.

## Data availability statement

The datasets generated during and/or analyzed during the current study are available from the corresponding author on reasonable request.

## Ethics statement

The studies involving humans were approved by Ethics Committee of the Japan Conference of Clinical Research (a non-profit organization). The studies were conducted in accordance with the local legislation and institutional requirements. Written informed consent for participation in this study was provided by the participants’ legal guardians/next of kin.

## Author contributions

All authors contributed to the article and approved the submitted version. HK, MI, TK, NO, MG, and SN were involved in the study design. HK, MI, TK, and SN were advisors in the study. HK, MI, and TK were involved in data collection.

## References

[1] Seidel MG Kindle G Gathmann B Quinti I Buckland M van Montfrans J. The European Society for Immunodeficiencies (ESID) Registry working definitions for the clinical diagnosis of inborn errors of immunity. J Allergy Clin Immunol Pract (2019) 7(6):1763–70. doi: 10.1016/j.jaip.2019.02.004 30776527

[2] Tangye SG Al-Herz W Bousfiha A Cunningham-Rundles C Franco JL Holland SM. Human inborn errors of immunity: 2022 update on the classification from the International Union of Immunological Societies Expert Committee. J Clin Immunol (2022) 42(7):1473–507. doi: 10.1007/s10875-022-01289-3 PMC9244088 35748970

[3] Cunningham-Rundles C. Key aspects for successful immunoglobulin therapy of primary immunodeficiencies. Clin Exp Immunol (2011) 164 (Suppl 2):16–9. doi: 10.1111/j.1365-2249.2011.04390.x PMC3087906 21466548

[4] McCusker C Upton J Warrington R. Primary immunodeficiency. Allergy Asthma Clin Immunol (2018) 14(Suppl 2):61. doi: 10.1186/s13223-018-0290-5 30275850 PMC6157160

[5] Tangye SG Al-Herz W Bousfiha A Chatila T Cunningham-Rundles C Etzioni A. Human inborn errors of immunity: 2019 update on the classification from the International Union of Immunological Societies Expert Committee. J Clin Immunol (2020) 40(1):24–64. doi: 10.1007/s10875-019-00737-x 31953710 PMC7082301

[6] Hosaka S Kido T Imagawa K Fukushima H Morio T Nonoyama S. Vaccination for patients with inborn errors of immunity: a nationwide survey in Japan. J Clin Immunol (2022) 42(1):183–94. doi: 10.1007/s10875-021-01160-x 34704141

[7] Ishimura M Takada H Doi T Imai K Sasahara Y Kanegane H. Nationwide survey of patients with primary immunodeficiency diseases in Japan. J Clin Immunol (2011) 31(6):968–76. doi: 10.1007/s10875-011-9594-7 21956496

[8] Berg AK Diseth TH Abrahamsen TG Halvorsen K Reinfjell T Erichsen HC. Primary antibody deficiency: the impact on the quality of life and mental health of affected children and their parents. Acta Paediatr (2021) 110(5):1645–52. doi: 10.1111/apa.15752 33420742

[9] Espanol T Prevot J Drabwell J Sondhi S Olding L. Improving current immunoglobulin therapy for patients with primary immunodeficiency: quality of life and views on treatment. Patient Prefer Adherence (2014) 8:621–9. doi: 10.2147/ppa.S60771 PMC4014377 24833896

[10] Jiang F Torgerson TR Ayars AG. Health-related quality of life in patients with primary immunodeficiency disease. Allergy Asthma Clin Immunol (2015) 11:27. doi: 10.1186/s13223-015-0092-y 26421019 PMC4587876

[11] Peshko D Kulbachinskaya E Korsunskiy I Kondrikova E Pulvirenti F Quinti I. Health-related quality of life in children and adults with primary immunodeficiencies: a systematic review and meta-analysis. J Allergy Clin Immunol Pract (2019) 7(6):1929–57.e5. doi: 10.1016/j.jaip.2019.02.013 30797077

[12] EFPIA-Japan. Primary immunodeficiency (PID). Survey on the treatment and quality of life of patients. Results of the questionnaire survey (2018). Available at: http://efpia.jp/link/PID_E_190130.pdf (Accessed January 23, 2023).

[13] Grassly NC Fraser C. Seasonal infectious disease epidemiology. Proc Biol Sci (2006) 273(1600):2541–50. doi: 10.1098/rspb.2006.3604 PMC1634916 16959647

[14] Hibiya K Iwata H Kinjo T Shinzato A Tateyama M Ueda S. Incidence of common infectious diseases in Japan during the COVID-19 pandemic. PloS One (2022) 17(1):e0261332. doi: 10.1371/journal.pone.0261332 35020724 PMC8754328

[15] Moriyama M Hugentobler WJ Iwasaki A. Seasonality of respiratory viral infections. Annu Rev Virol (2020) 7(1):83–101. doi: 10.1146/annurev-virology-012420-022445 32196426

[16] Herdman M Gudex C Lloyd A Janssen MF Kind P Parkin D. Development and preliminary testing of the new five-level version of EQ-5D (EQ-5D-5L). Qual Life Res (2011) 20(10):1727–36. doi: 10.1007/s11136-011-9903-x PMC3220807 21479777

[17] Ware JE Jr Sherbourne CD. The MOS 36-item short-form health survey (SF-36). I. Conceptual framework and item selection. Med Care (1992) 30(6):473–83. doi: 10.1097/00005650-199206000-00002 1593914

[18] Shiroiwa T Fukuda T Ikeda S Igarashi A Noto S Saito S. Japanese population norms for preference-based measures: EQ-5D-3L, EQ-5D-5L, and SF-6D. Qual Life Res (2016) 25(3):707–19. doi: 10.1007/s11136-015-1108-2 PMC4759213 26303761

[19] Fukuhara S Bito S Green J Hsiao A Kurokawa K. Translation, adaptation, and validation of the SF-36 Health Survey for use in Japan. J Clin Epidemiol (1998) 51(11):1037–44. doi: 10.1016/s0895-4356(98)00095-x 9817121

[20] Reilly MC Zbrozek AS Dukes EM. The validity and reproducibility of a work productivity and activity impairment instrument. Pharmacoeconomics (1993) 4(5):353–65. doi: 10.2165/00019053-199304050-00006 10146874

[21] Gathmann B Mahlaoui N Gérard L Oksenhendler E Warnatz K Schulze I. Clinical picture and treatment of 2212 patients with common variable immunodeficiency. J Allergy Clin Immunol (2014) 134(1):116–26. doi: 10.1016/j.jaci.2013.12.1077 24582312

[22] Immune Deficiency Foundation. Primary immune deficiency diseases in America: 2007 the third national survey of patients (2007). Available at: https://primaryimmune.org/publication/surveys/primary-immune-deficiency-diseases-america-third-national-survey-patients-2007 (Accessed May 28, 2023).

[23] Slade CA Bosco JJ Binh Giang T Kruse E Stirling RG Cameron PU. Delayed diagnosis and complications of predominantly antibody deficiencies in a cohort of Australian adults. Front Immunol (2018) 9:694. doi: 10.3389/fimmu.2018.00694 29867917 PMC5960671

[24] Imai K Oh A Morishita A Inoue Y. Physician awareness and understanding of primary immunodeficiency disorders: a web-based study in Japan. Immunol Med (2023) 46(1):45–57. doi: 10.1080/25785826.2022.2137966 36330855

[25] Arslan S Ucar R Caliskaner AZ Reisli I Guner SN Sayar EH. How effective are the 6 European Society of Immunodeficiency warning signs for primary immunodeficiency disease? Ann Allergy Asthma Immunol (2016) 116(2):151–5.e1. doi: 10.1016/j.anai.2015.12.001 26815708

[26] Epland K Suez D Paris K. A clinician’s guide for administration of high-concentration and facilitated subcutaneous immunoglobulin replacement therapy in patients with primary immunodeficiency diseases. Allergy Asthma Clin Immunol (2022) 18(1):87. doi: 10.1186/s13223-022-00726-7 36180928 PMC9526304

[27] Japanese Society for Immunodeficiencies. Clinical practice guide for patients with primary immunodeficiency syndrome [Japanese]. Tokyo: Shindan to Chiryo sha (2017).

[28] Shehata N Palda V Bowen T Haddad E Issekutz TB Mazer B. The use of immunoglobulin therapy for patients with primary immune deficiency: an evidence-based practice guideline. Transfus Med Rev (2010) 24 Suppl 1:S28–50. doi: 10.1016/j.tmrv.2009.09.011 19962579

[29] Orange JS Grossman WJ Navickis RJ Wilkes MM. Impact of trough IgG on pneumonia incidence in primary immunodeficiency: a meta-analysis of clinical studies. Clin Immunol (2010) 137(1):21–30. doi: 10.1016/j.clim.2010.06.012 20675197

[30] Lumry WR Castaldo AJ Vernon MK Blaustein MB Wilson DA Horn PT. The humanistic burden of hereditary angioedema: impact on health-related quality of life, productivity, and depression. Allergy Asthma Proc (2010) 31(5):407–14. doi: 10.2500/aap.2010.31.3394 20929608

[31] Mendivil J Murphy R de la Cruz M Janssen E Boysen HB Jain G. Clinical characteristics and burden of illness in patients with hereditary angioedema: findings from a multinational patient survey. Orphanet J Rare Dis (2021) 16(1):94. doi: 10.1186/s13023-021-01717-4 33602292 PMC7893968

