# Supplementary material for: Patient-reported outcomes in patients with primary immunodeficiency diseases in Japan: baseline results from a prospective observational study
Source: Front Immunol. 2023 Sep 27;14:1244250. doi: 10.3389/fimmu.2023.1244250 (PMC10565343; doi:10.3389/fimmu.2023.1244250)

Supplementary Material

**Patient-Reported Outcomes in Patients with Primary Immunodeficiency Diseases in Japan: Baseline Results from a Prospective Observational Study**

Hirokazu Kanegane^*^, Masataka Ishimura, Toshinao Kawai, Satoshi Okada, Nobuaki Okamatsu, Madoka Go, Shinichi Noto

*** Correspondence:** Hirokazu Kanegane: hkanegane.ped@tmd.ac.jp

**Supplementary Table 1 Custom questions created for this study.**

| **Categories** | **Questions** |
| --- | --- |
| Demographics | How old are you? (years) |
|  | What is your gender? |
| Diagnosis | What is your diagnosis? |
|  | How old were you when you were diagnosed with primary immunodeficiency? |
|  | What event led to your diagnosis? |
|  | How long did it take to obtain a diagnosis for your condition after the onset of symptoms? (months) |
| Complications | What chronic complications do you have now or did you have previously? (multiple responses possible) |
| Infections | What types of infection did you contract in the past 5 years? (multiple responses possible) |
|  | How many times did you have an infection in the past 5 years? |
|  | How many times were you hospitalized for an infection in the past 5 years? |
| Treatments | What type of treatment are you currently receiving for primary immunodeficiency? (multiple responses possible) |
|  | Do you know what your serum IgG level was before the initial dose of immunoglobulin? |
|  | Is your serum IgG level measured routinely on the day that immunoglobulin is prescribed? |
|  | What was your serum IgG level immediately before the last immunoglobulin prescription (in mg/dL)? |
| Work and school status | What is your current status regarding work/school? |
|  | Please give the reason for your current work status. |
|  | At what age did you resign or take a leave of absence from your job? (years) |
|  | How many months have passed since you resigned or took leave of absence? (months) |
|  | What kind of work-related difficulties have you experienced from having primary immunodeficiency? (multiple responses possible) |
|  | What are your current thoughts about employment? |
| Visits to medical institutions | Regarding your routine visits for primary immunodeficiency, please tell us how many medical institutions/ departments you visit. |
|  | How often do you make routine visits for primary immunodeficiency? (single response for each institution) |
|  | Do you find it difficult to make time for routine visits? |
|  | What is the longest time you spend at the medical institution for your routine visit? (if you make routine visits to more than one medical institution, please select the longest time you spend at an institution) |
|  | What is the longest time it takes to travel (one way) to a medical institution for your routine visit? (if you make routine visits to more than one medical institution, please select the longest time you travel) |
|  | What is the maximum cost of a round trip (public transportation or taxi) for your routine visit? |
|  | Which medical department do you visit for primary immunodeficiency? (multiple responses possible) |
|  | Is your attending doctor a specialist in primary immunodeficiency (a doctor at a university hospital or another large medical institution)? |
|  | What type of medical institution do you visit in case of acute symptoms such as fever? (multiple responses possible) |
| Social support and expenses | Do you have a physical disability certification? |
|  | For which disability did you receive the certification? (multiple responses possible) |
|  | Do you receive public assistance (for a designated intractable disease or specific pediatric chronic disease, etc.) for expenses related to medical care? |
|  | Do you feel that the medical expenses for treatment of primary immunodeficiency (including complications) are a financial burden? |
|  | Do you feel that physical disability certification is necessary to reduce the financial burden of medical expenses? |
|  | Do you feel that physical disability certification is necessary to reduce expenses for travelling long distances to visit a specialist (ie, discounted fares for public transportation)? |
|  | Do you feel that physical disability certification is necessary to expand opportunities for work, such as obtaining regular employment contracts or a job that matches your qualifications, within the employment framework for persons with disabilities? |
|  | Do you feel that physical disability certification is necessary to allow you to work with appropriate workplace considerations within the employment framework for persons with disabilities? |
| Limitations in daily life | Which symptoms of primary immunodeficiency impose limitations in your daily life?  Select the items that apply to the past 3 months. (multiple responses possible) |
|  | How is your daily life limited by having to receive various types of medical care?  Select the items that apply to the past 3 months. (multiple responses possible) |
|  | What measures do you take to prevent symptoms?  Select the items that apply to the past 3 months. (multiple responses possible) |

IgG, immunoglobulin G.

**Supplementary Figure 1.** Medical visit data in patients with primary immunodeficiency disease (n=71). **(A)** Number of medical institutions visited. **(B)** Frequency of medical visits (multiple responses possible; n=82 responses). **(C)** Time burden of visits. **(D)** Time spent at the medical institution. **(E)** Time spent traveling for the medical visit. **(F)** Transportation cost per visit. **(G)** Medical department visited (multiple responses possible; n=158 responses). **(H)** Attending doctor. **(I)** Medical institution visited when acute symptoms occur (multiple responses possible; n=92 responses).

**(A)**

**
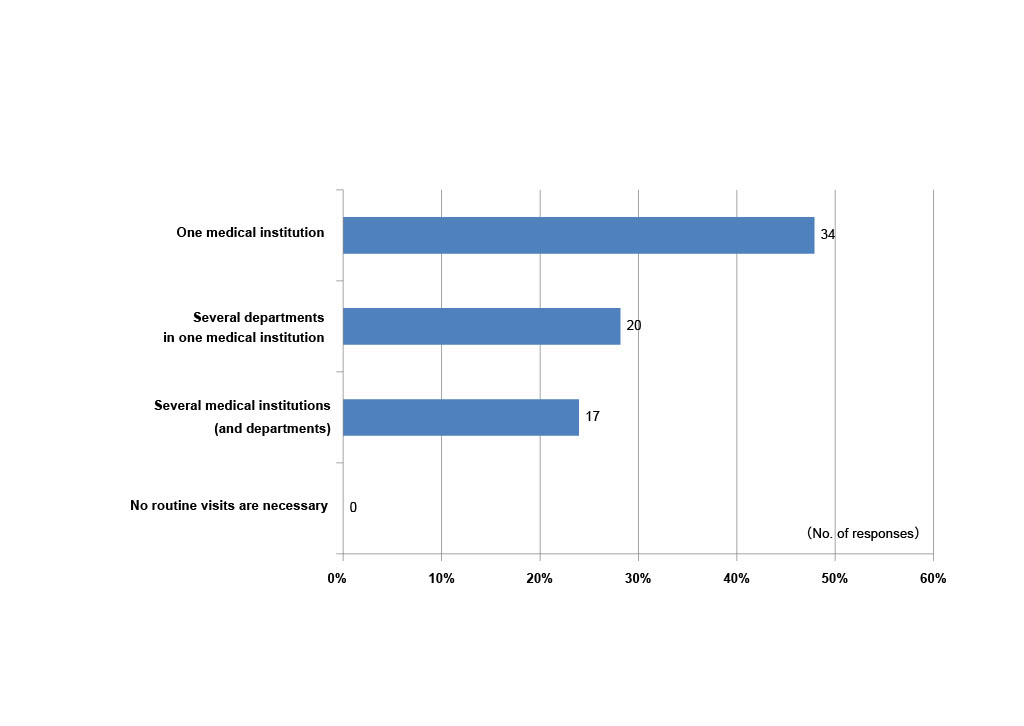
**

**(B)**

**
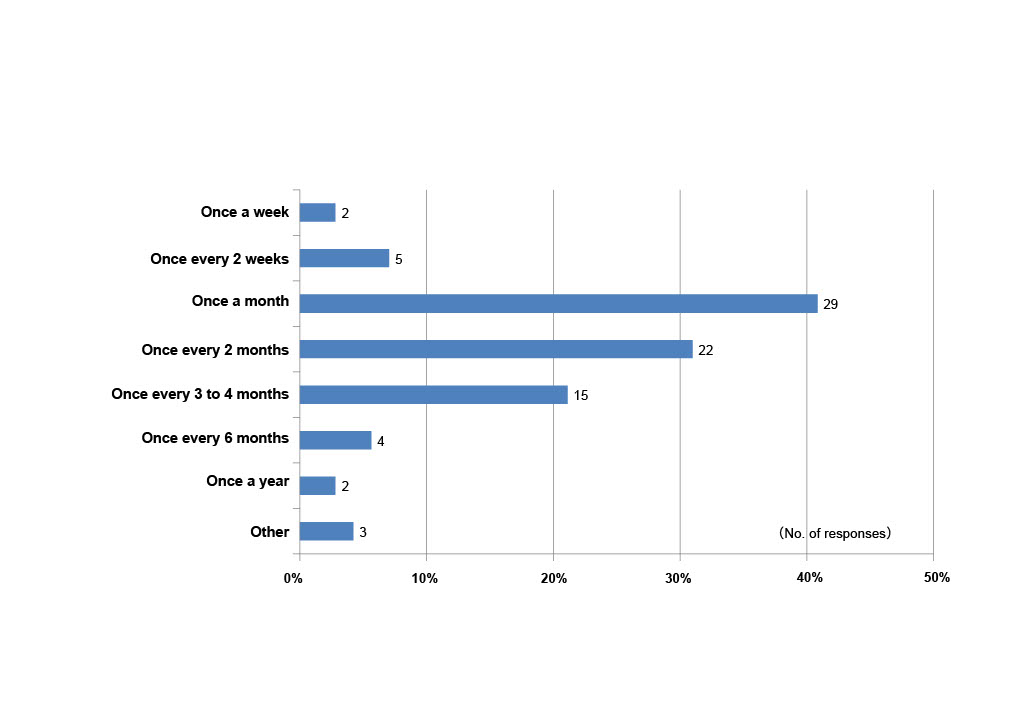
**

**(C)**

**
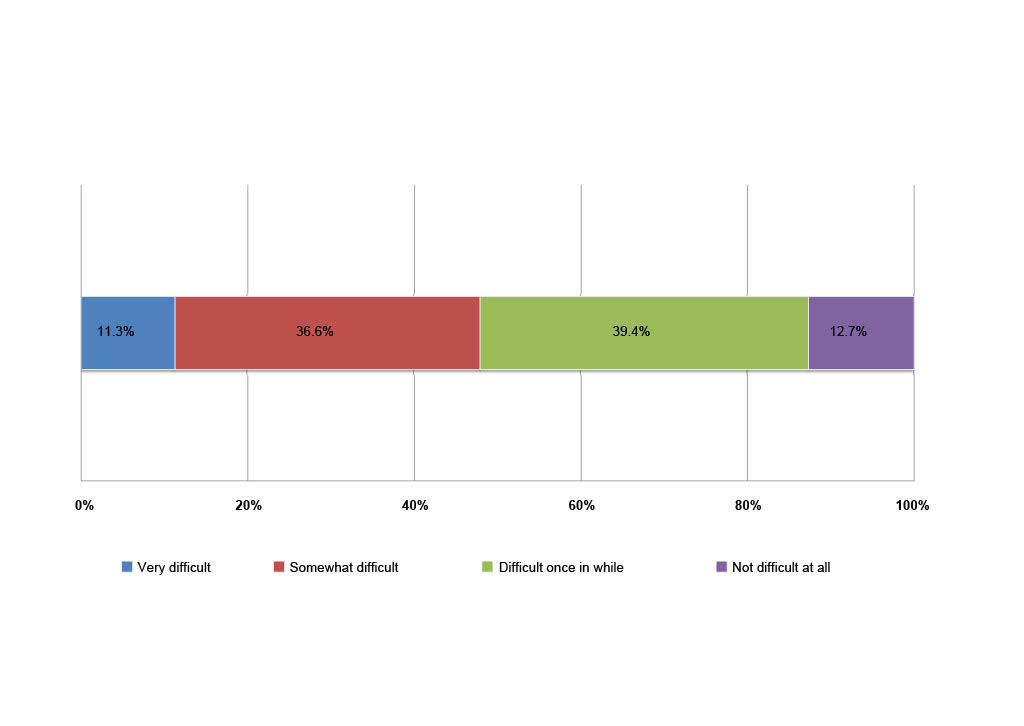
**

**(D)**

**
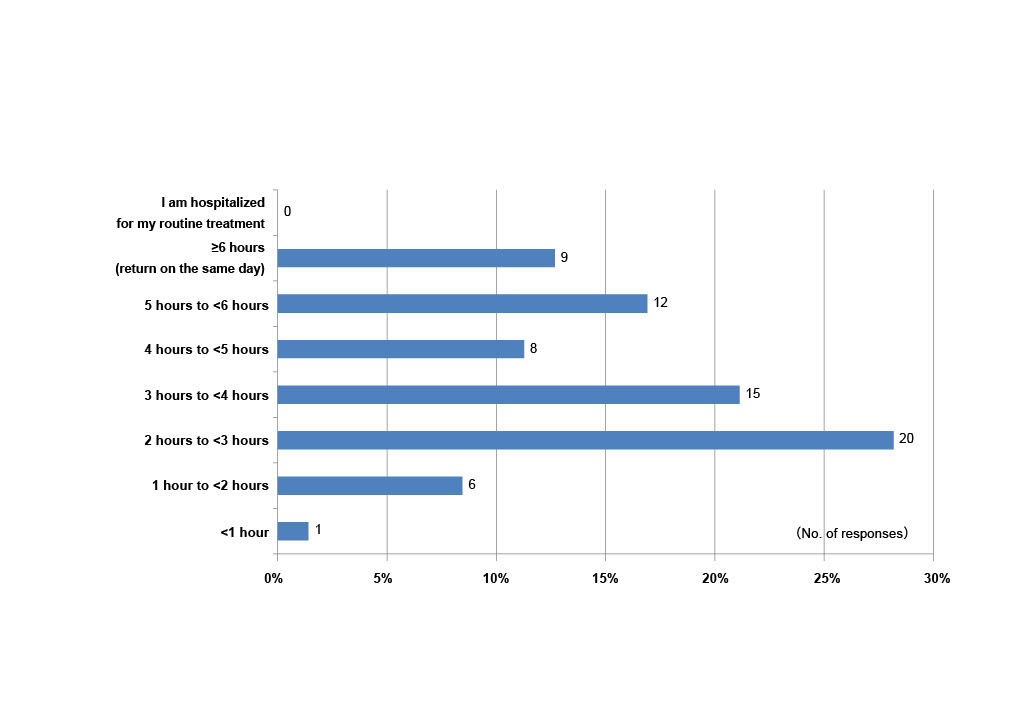
**

**(E)**

**
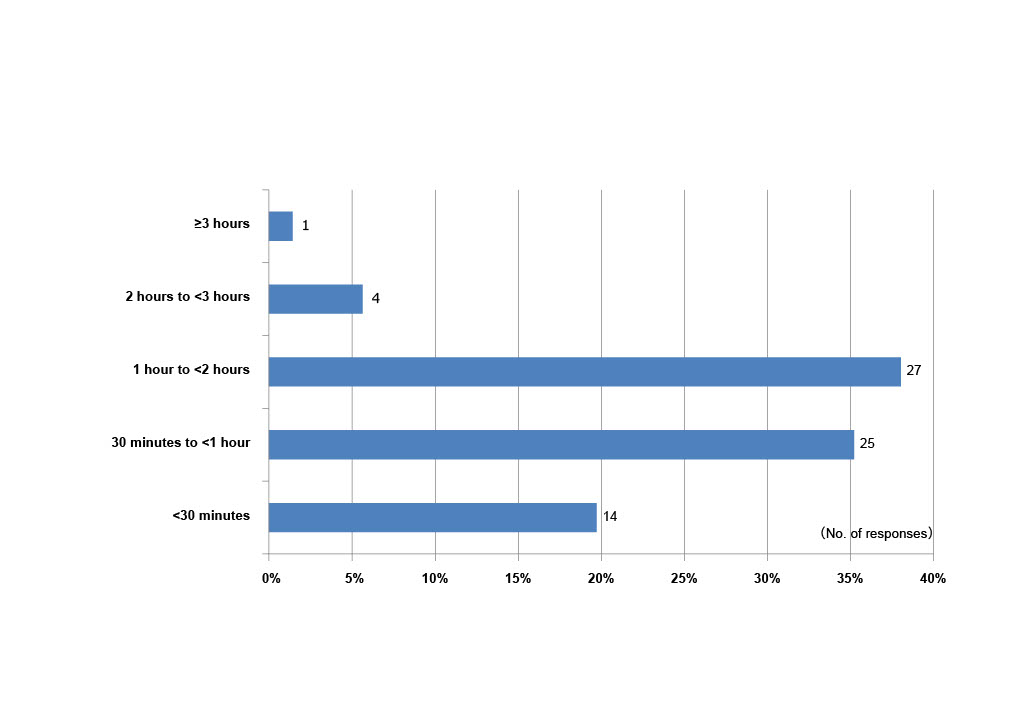
**

**(F)**

**
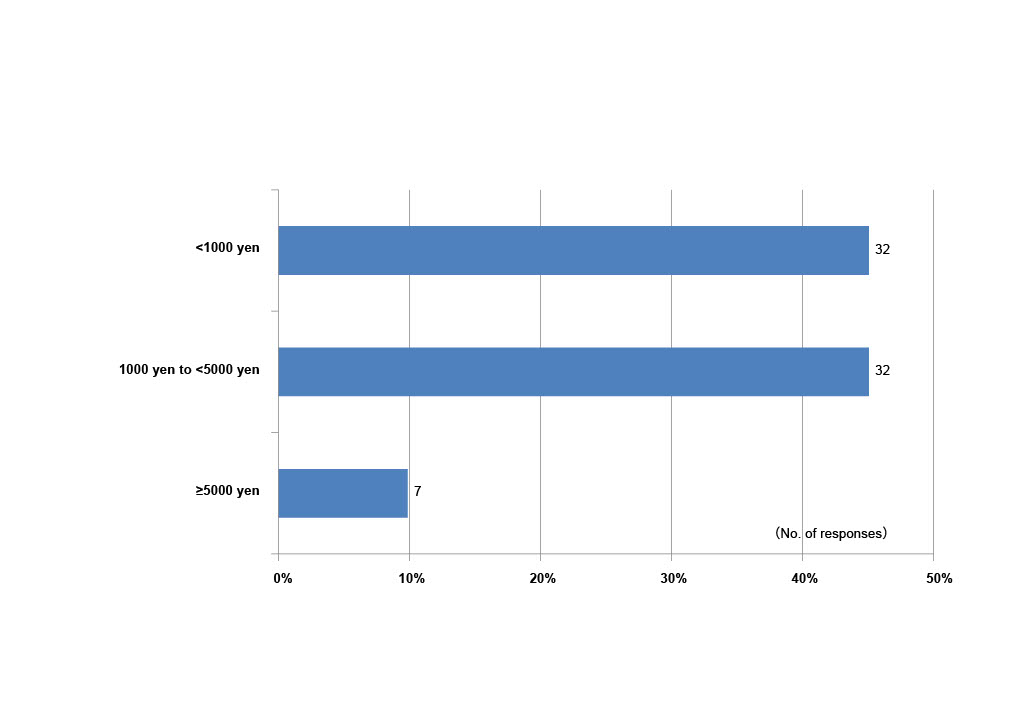
**

**(G)**

**
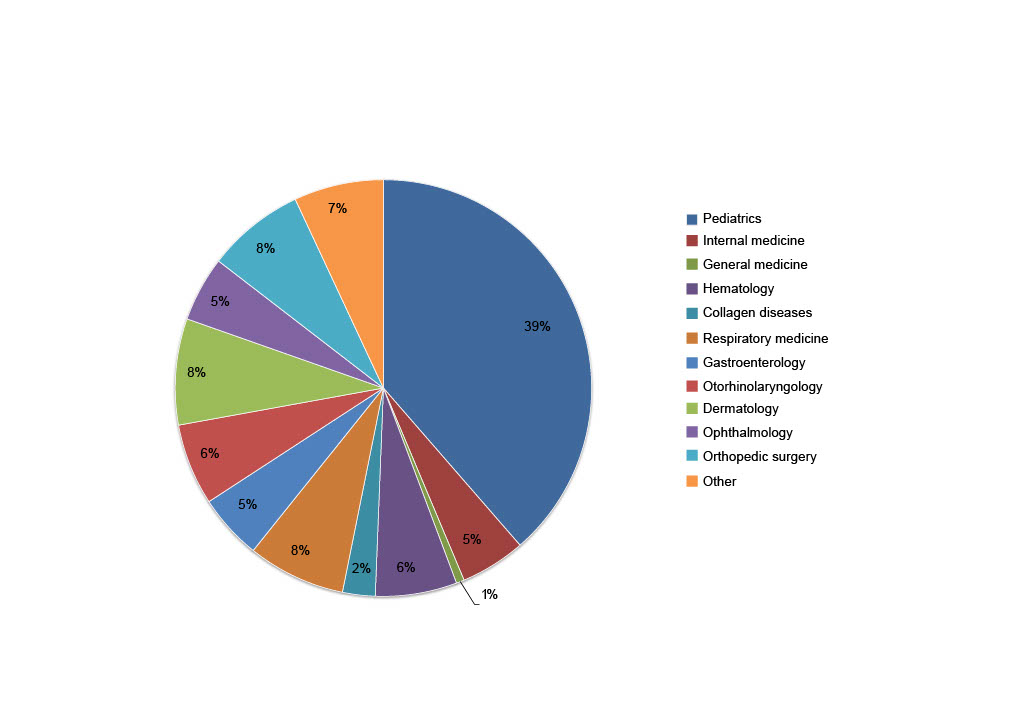
**

**(H)**

**
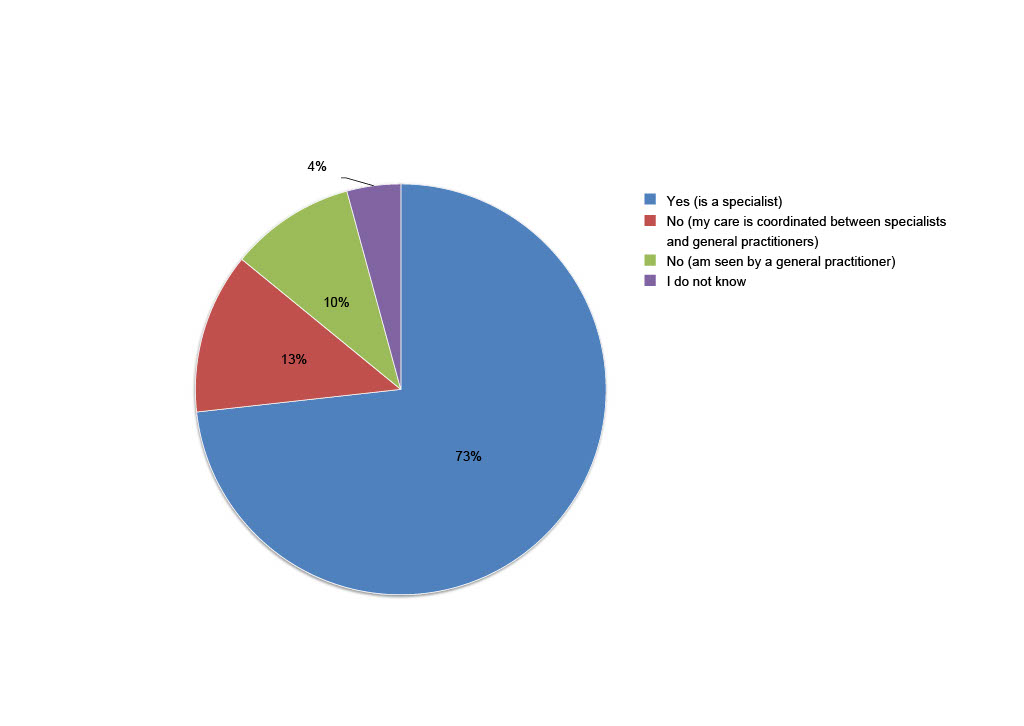
**

**(I)**

**
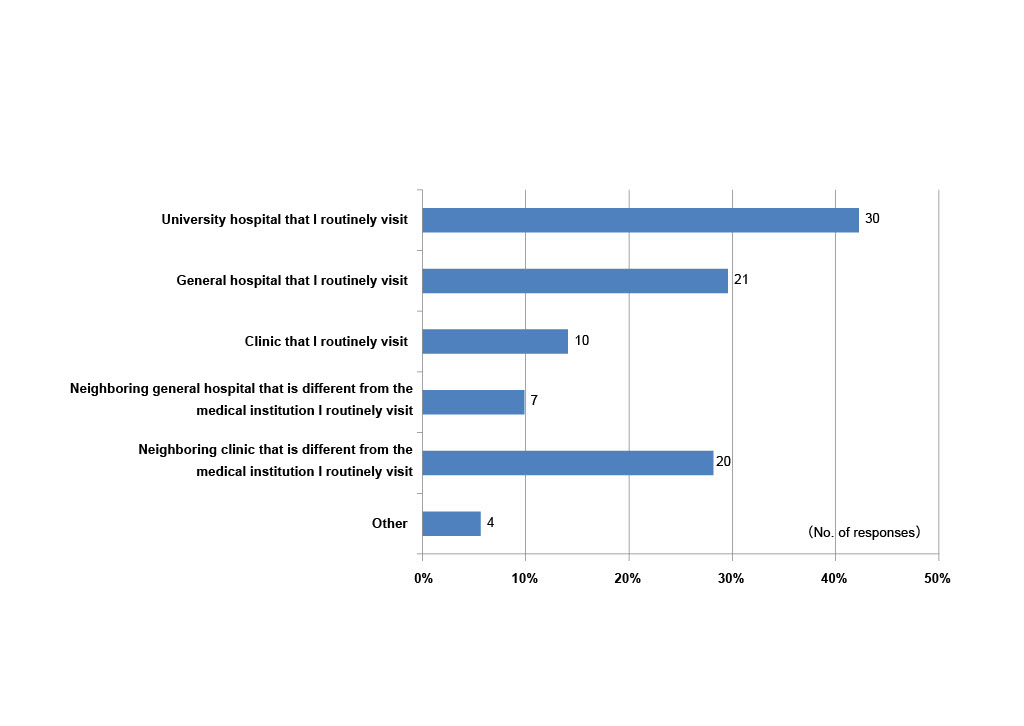
**

**Supplementary Figure 2.** Expenses and social support in patients with primary immunodeficiency disease (n=71). **(A)** Burden of expenses. **(B)** Public assistance for expenses related to medical care. **(C)** Necessity of physical disability certification. **(D)** Physical disability certification. **(E)** Reason for certification (n=9 patients with certification; multiple responses possible, n=11 responses).

**(A)**

**
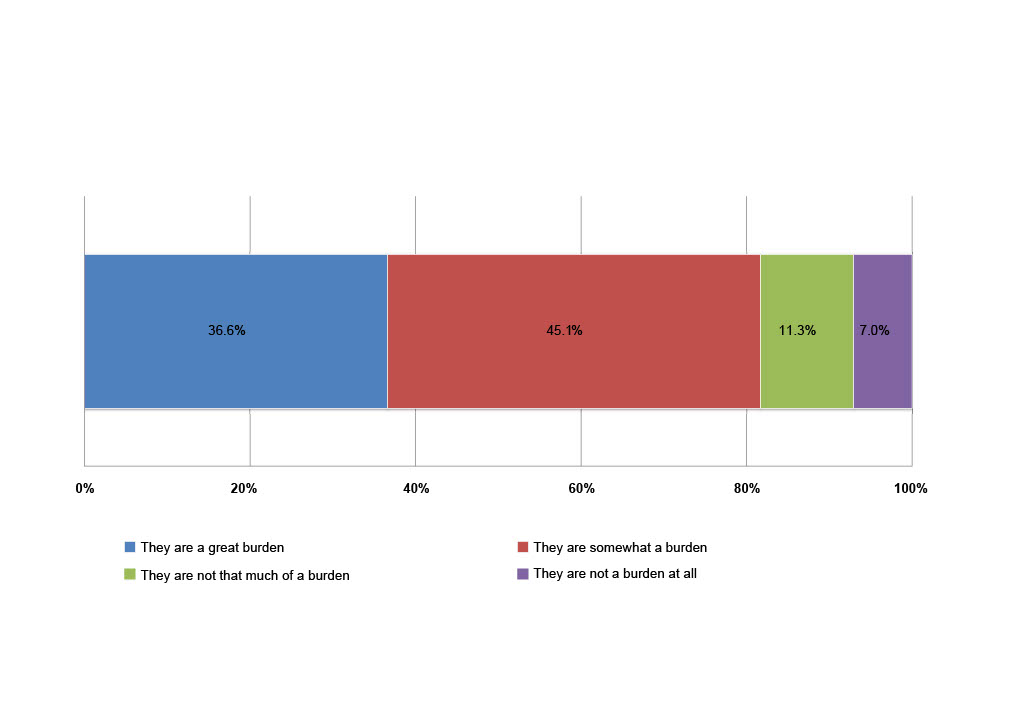
**

**(B)**

**
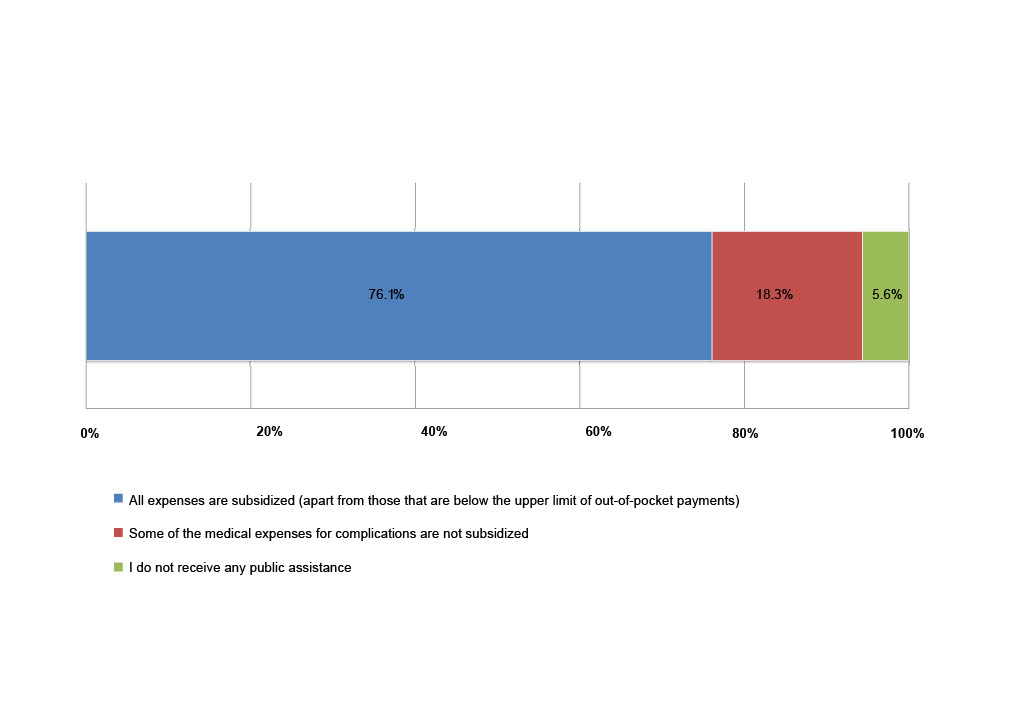
**

**(C)**

**
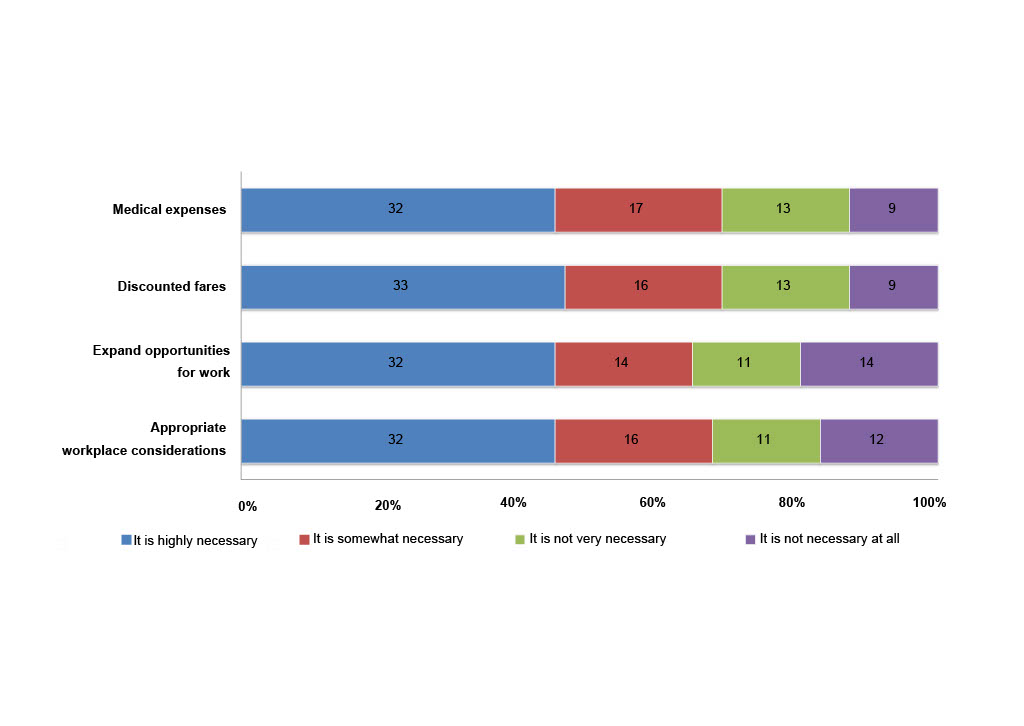
**

**(D)**

**
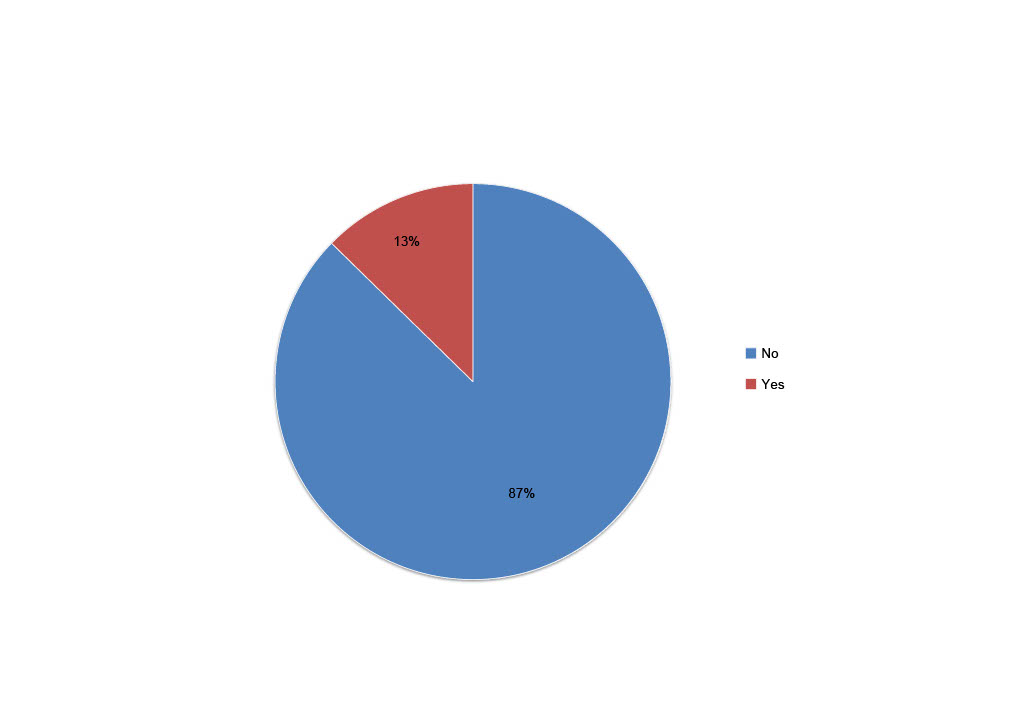
**

**(E)**

**
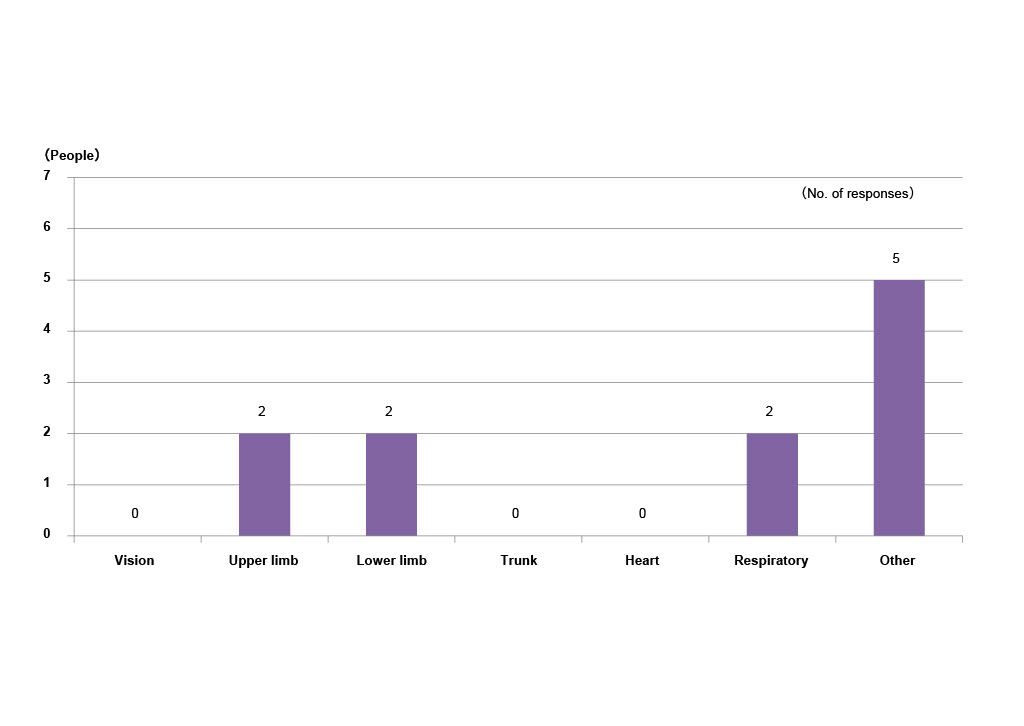
**

**Supplementary Figure 3.** Work and school status in patients with primary immunodeficiency disease (n=71).

**
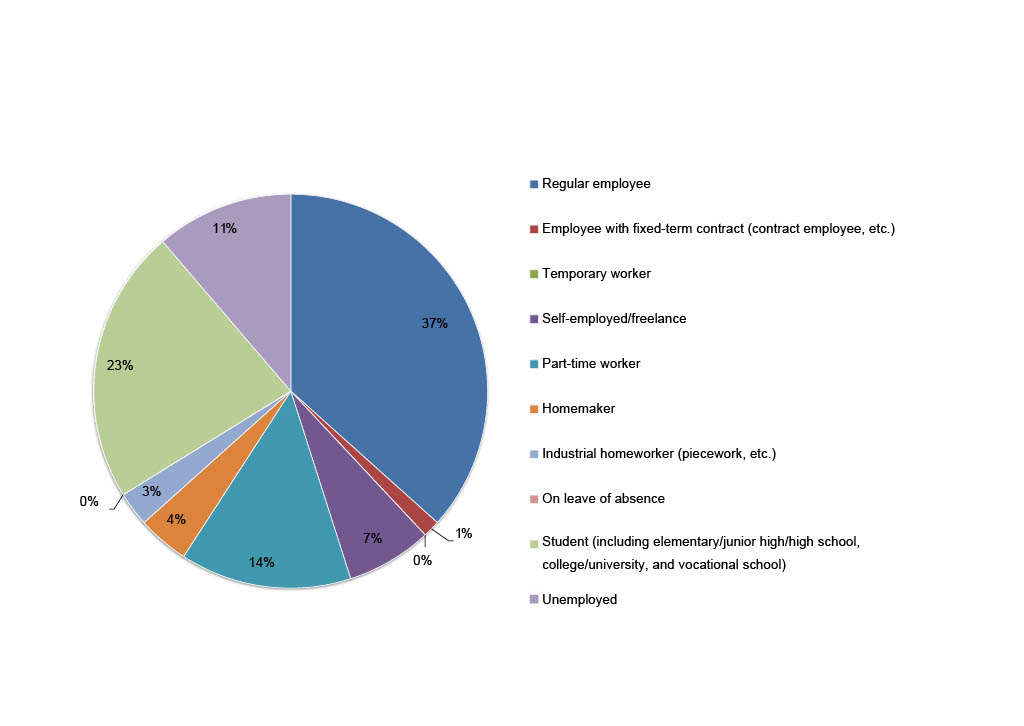
**

**Supplementary Figure 4.** Work-related data in patients with primary immunodeficiency disease who are not students (n=55). **(A)** Current employment status. **(B)** Thoughts about employment. **(C)** Reason for current work status in patients with primary immunodeficiency disease whose current work status is homemaker, industrial homeworker (eg, piecework), on leave, or unemployed; n=13).

**(A)**


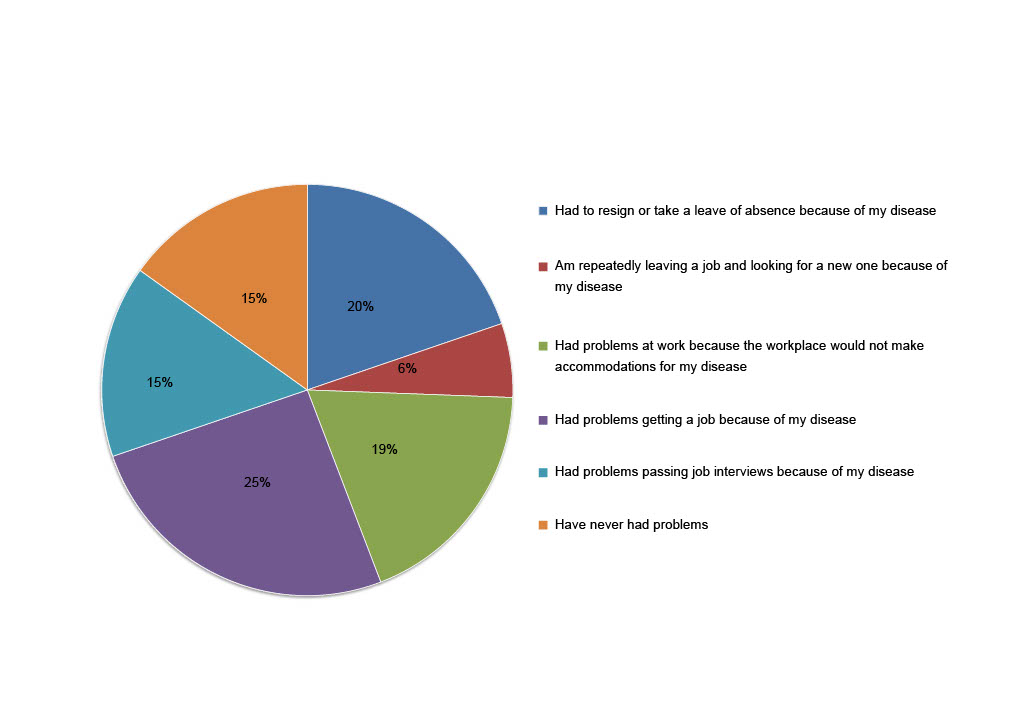


**(B)**

**
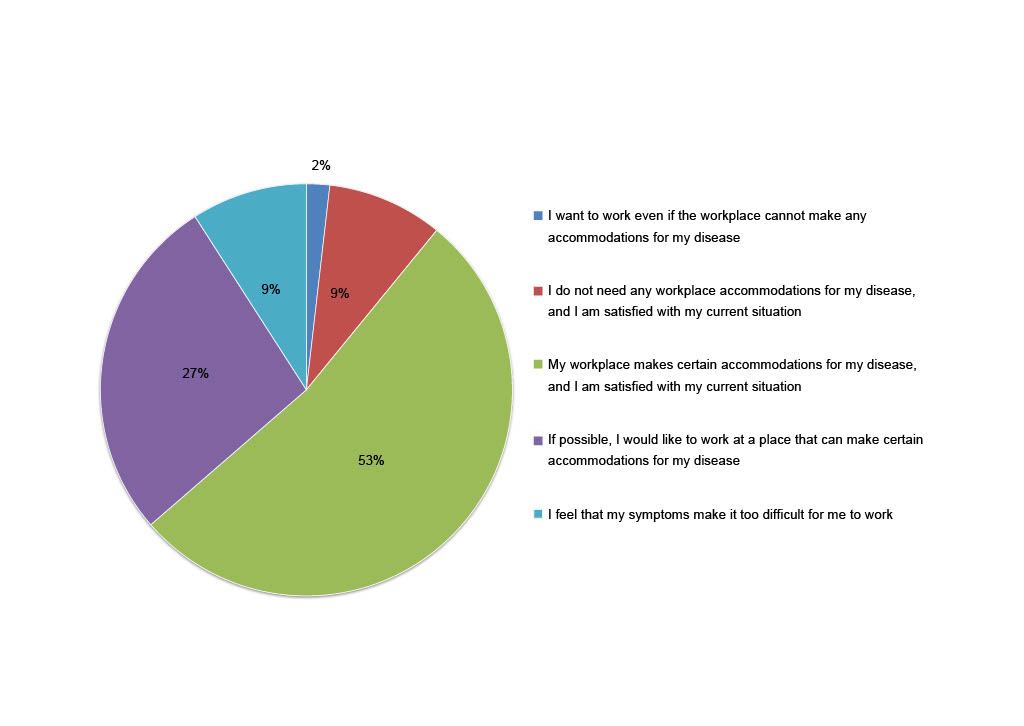
**

**(C)**

**
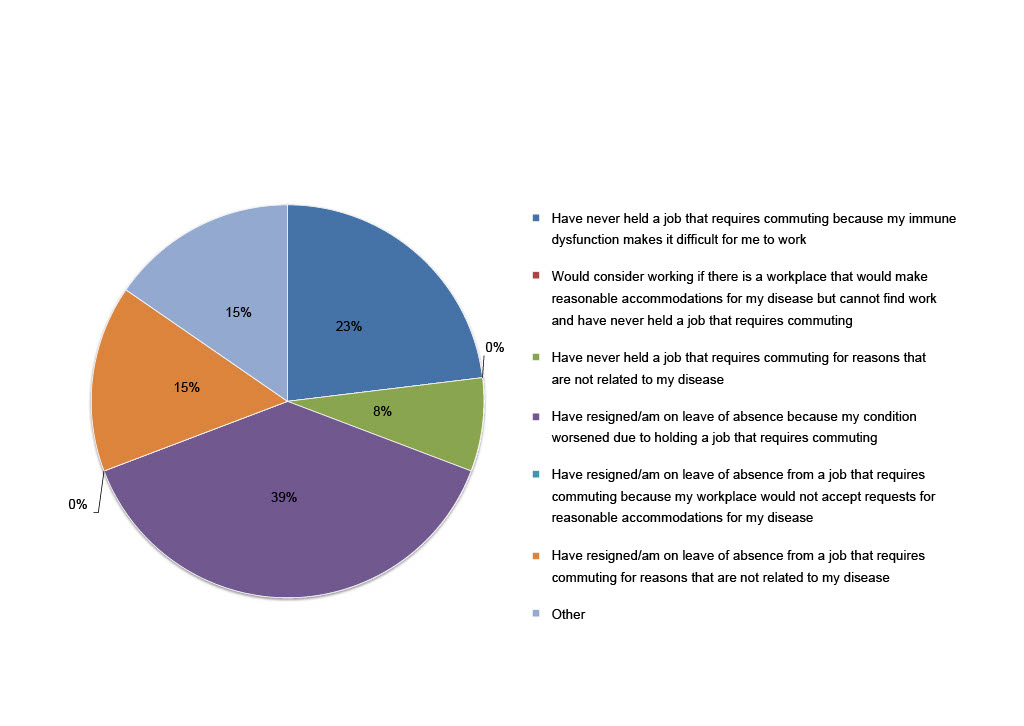
**

**Supplementary Figure 5.** SF-36 subscale scores in patients with PID (non-HCT subgroup) compared with healthy individuals (PPS). *p < 0.05; ***p ≤ 0.001. BP, Bodily Pain; GH, General Health; HCT, hematopoietic cell transplantation; MH, Mental Health; ns, not significant; PF, Physical Functioning; PID, primary immunodeficiency disease; PPS, per-protocol set; RE, Role-Emotional; RP, Role-Physical; SD, standard deviation; SF, Social Functioning; SF-36, 36-Item Short Form Health Survey; VT, Vitality.


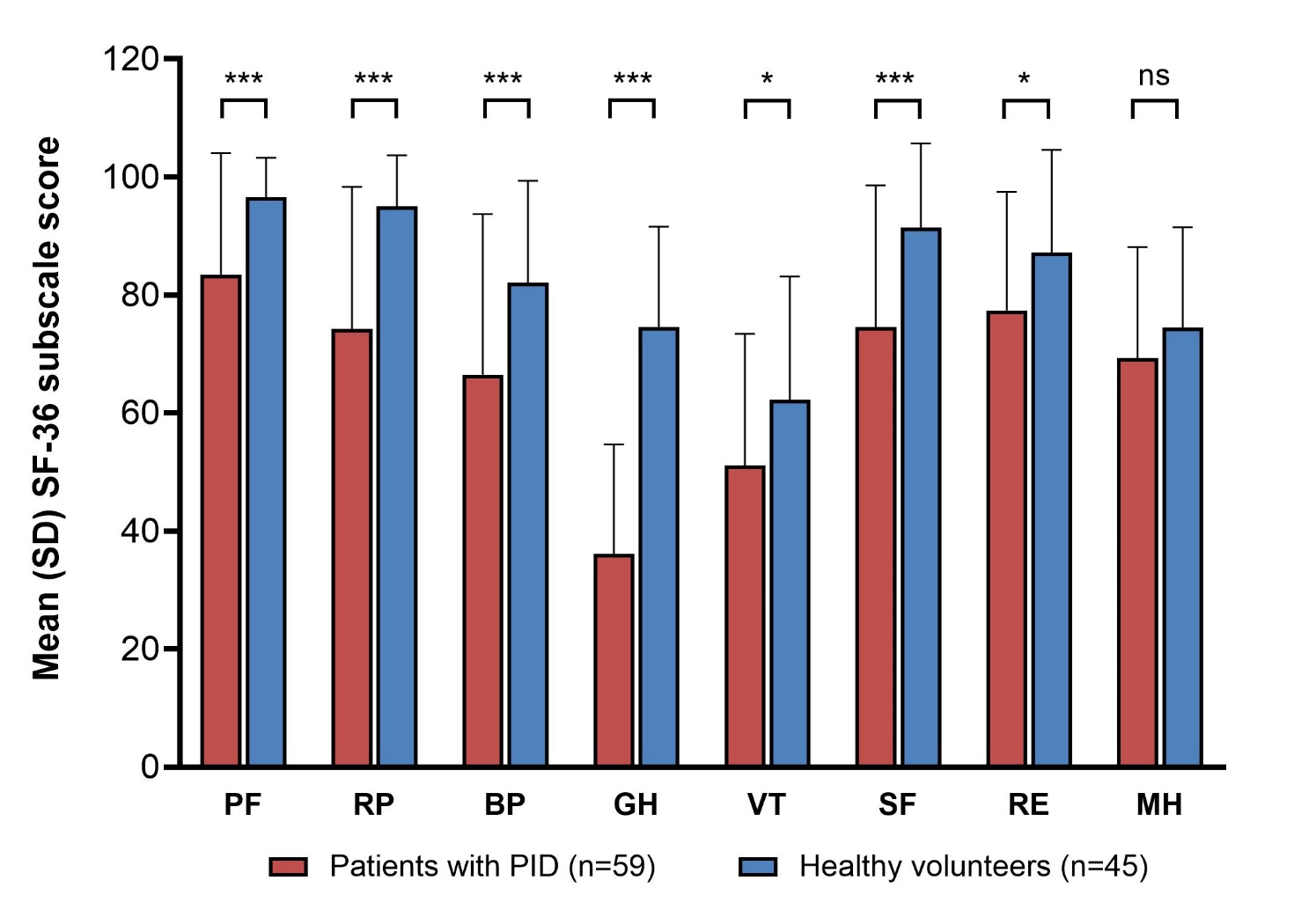


**Supplementary Figure 6.** WPAI in individual patients with PID (FAS/PPS; n=44) compared with healthy individuals (PPS; n=37). **(A)** Absenteeism, **(B)** Presenteeism, **(C)** Work Productivity Loss, **(D)** Activity Impairment. Lines represent medians; error bars represent interquartile ranges. p-Values are from the Mann-Whitney U test comparing between groups. FAS, full analysis set; PID, primary immunodeficiency disease; PPS, per-protocol set; WPAI, Work Productivity and Activity Impairment.


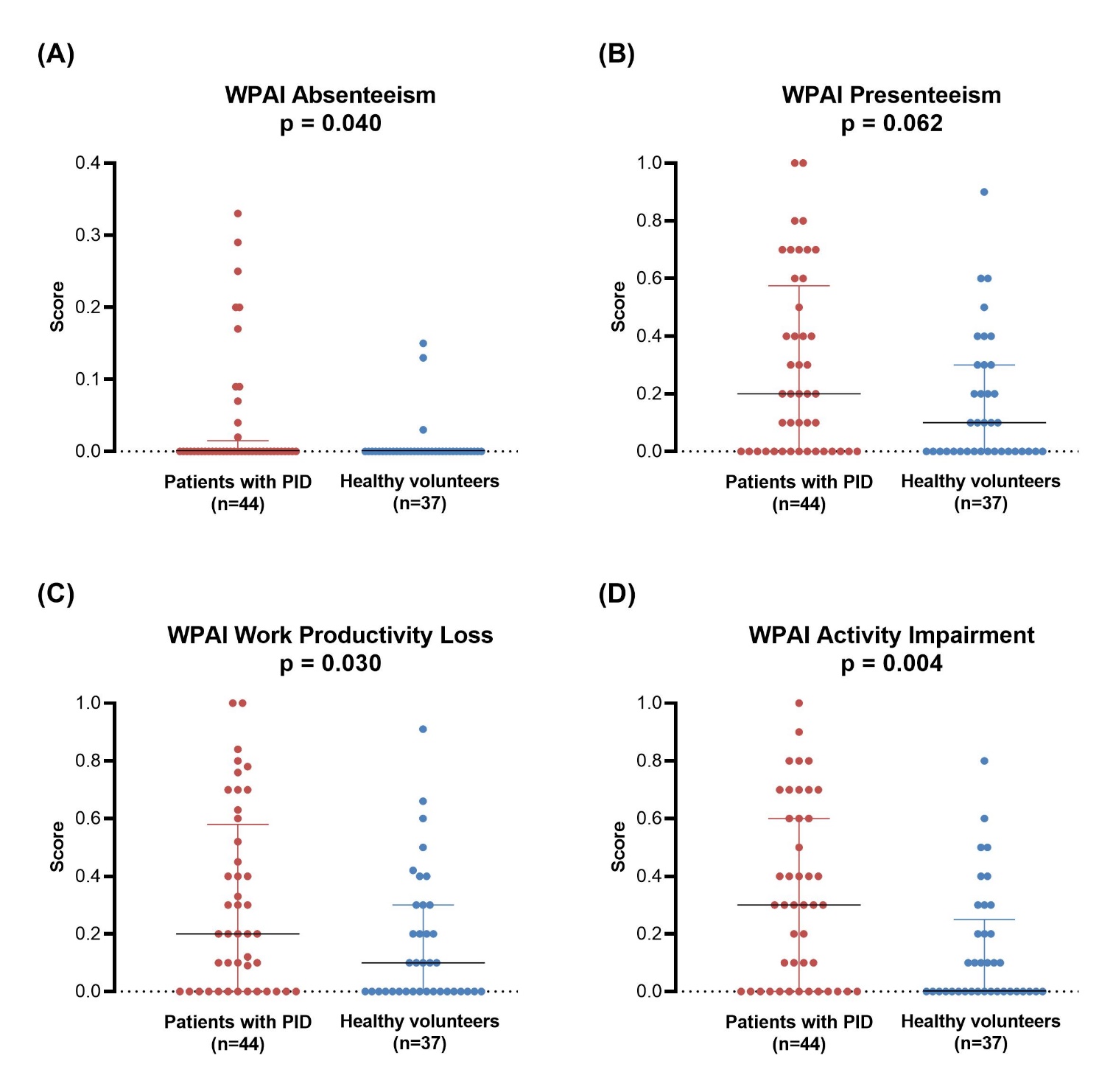

Supplement: Supplementary file 1 [file DataSheet_1.docx]
